# TGF‐β‐induced PI3K/AKT/mTOR pathway controls myofibroblast differentiation and secretory phenotype of valvular interstitial cells through the modulation of cellular senescence in a naturally occurring in vitro canine model of myxomatous mitral valve disease

**DOI:** 10.1111/cpr.13435

**Published:** 2023-03-04

**Authors:** Qiyu Tang, Greg R. Markby, Andrew J. MacNair, Keyi Tang, Michal Tkacz, Maciej Parys, Kanchan Phadwal, Vicky E. MacRae, Brendan M. Corcoran

**Affiliations:** ^1^ The Roslin Institute The University of Edinburgh Edinburgh UK; ^2^ Royal (Dick) School of Veterinary Studies The University of Edinburgh Edinburgh UK

## Abstract

PI3K/AKT/mTOR signalling contributes to several cardiovascular disorders. The aim of this study was to examine the PI3K/AKT/mTOR pathway in myxomatous mitral valve disease (MMVD). Double‐immunofluorescence examined expression of PI3K and TGF‐β1 in canine valves. Valve interstitial cells (VICs) from healthy or MMVD dogs were isolated and characterized. Healthy quiescent VICs (qVICs) were treated with TGF‐β1 and SC‐79 to induce activated myofibroblast phenotypes (aVICs). Diseased valve‐derived aVICs were treated with PI3K antagonists and expression of RPS6KB1 (encoding p70 S6K) was modulated using siRNA and gene overexpression. SA‐β‐gal and TUNEL staining were used to identify cell senescence and apoptosis, and qPCR and ELISA to examine for senescence‐associated secretory phenotype. Protein immunoblotting was used to examine expression of phosphorylated and total proteins. TGF‐β1 and PI3K are highly expressed in mitral valve tissues. Activation of PI3K/AKT/mTOR and increased expression of TGF‐β are found in aVICs. TGF‐β transitions qVICs to aVICs by upregulation of PI3K/AKT/mTOR. Antagonism of PI3K/AKT/mTOR reverses aVIC myofibroblast transition by inhibiting senescence and promoting autophagy. Upregulation of mTOR/S6K induces transformation of senescent aVICs, with reduced capacity for apoptosis and autophagy. Selective knockdown of p70 S6K reverses cell transition by attenuating cell senescence, inhibiting apoptosis and improving autophagy. TGF‐β‐induced PI3K/AKT/mTOR signalling contributes to MMVD pathogenesis and plays crucial roles in the regulation of myofibroblast differentiation, apoptosis, autophagy and senescence in MMVD.

## Graphical abstract

Schematic illustration of TGF‐β‐induced PI3K/AKT/mTOR/p70 S6K pathway in the regulation of cell phenotype, extracellular matrix synthesis, apoptosis, autophagy and senescence in activated myofibroblasts in canine myxomatous mitral valve disease.

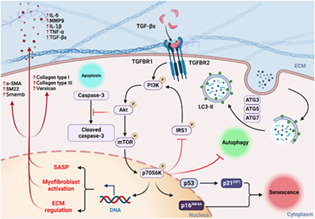

## 1 INTRODUCTION

Myxomatous mitral valve disease (MMVD) is one of the most devastating heart valve diseases in humans (syndromic and non‐syndromic forms) and dogs and a major cause of heart failure and sudden cardiac death, leading to significant morbidity and mortality in both species.^1^, ^2^, ^3^ It accounts for 7% of deaths in dogs before 10 years of age and its prevalence is estimated to be between 30% and 70% of all elderly dogs.^4^, ^5^ MMVD affects 2%–3% of the human global population with approximately 15% of those affected requiring surgical valve replacements.^6^ These treatments are invasive, costly, carry a risk for elderly adults and may lead to more severe complications including thrombosis, post‐operative infections and heart attack.^7^ Currently, there are no medications to prevent, slow progression or reverse valve pathology associated with MMVD. An improved understanding of the pathogenesis of MMVD is necessary for the development of novel therapeutic strategies for MMVD both in humans and dogs.

Accumulating evidence indicates that MMVD is a progressive and degenerative disease regulated by growth factors, in particular, members of the transforming growth factor β (TGF‐β) superfamily.^1^, ^8^ TGF‐β has been shown to have an important role in myxomatous degeneration in human MMVD and the associated end‐stage valve fibrosis.^9^, ^10^, ^11^, ^12^, ^13^ Aberrant upregulation of TGF‐β signalling has been reported in the various forms of mitral valve prolapse where myxomatous degeneration is found, including an X‐linked filamin‐A (FLNA) mutation, Marfan syndrome (MFS) and Barlow's Disease (BD).^1^ Similar observations are found in spontaneously occurring canine MMVD, although dogs lack end‐stage fibrosis, with transcriptomic data supporting the pivotal role of TGF‐β.^14^, ^15^ In human and canine myxomatous mitral valves, there is an increased number of valve interstitial cells (VICs) expressing α‐smooth muscle actin (α‐SMA), indicating an activated myofibroblast phenotype (aVICs).^16^, ^17^ TGF‐β has been shown to induce differentiation of cultured human and canine VICs to this myofibroblast phenotype, with associated excess extracellular matrix (ECM).^10^, ^18^, ^19^ Pharmacological antagonism of the TGF‐β receptor complex reverses aVICs back to a normal quiescent phenotype.^18^ Taken together, these studies indicate that the TGF‐β‐induced myofibroblast differentiation of VICs plays an important role in the pathogenesis of MMVD.

The role of canonical TGF‐β mediated Smad2/3 signalling in controlling VIC phenotype and ECM synthesis has been identified in human valve tissue, cultured primary VICs and transgenic mouse models.^9^, ^10^, ^11^, ^16^ However, a large‐scale clinical trial in children and young adults with MFS showed no effect by abolishing the Smad2/3 cascade using the angiotensin II receptor blocker losartan.^20^ This suggests that further investigation of the non‐canonical parts of the TGF‐β signalling pathway would be beneficial. Of particular interest is the phosphoinositide 3‐kinase (PI3K)/protein kinase B (AKT)/mammalian target of rapamycin (mTOR) pathway which is recognized to regulate multiple cellular processes including cell differentiation, survival and death.^21^ Dysregulation of PI3K/AKT/mTOR signalling is associated with a variety of degenerative disorders. In pulmonary fibrosis, TGF‐β‐induced differentiation of human lung fibroblasts to fibrogenic myofibroblasts is repressed by inhibiting the PI3K/AKT/mTOR pathway.^22^ The antagonism of PI3K/AKT/mTOR signalling has also been reported to promote autophagy of articular chondrocytes and attenuate the inflammatory response in rats with osteoarthritis.^23^ Recently, it has been shown that pharmacological inhibition and knockdown of mTOR/p70 S6 kinase (p70 S6K) signalling protects against intervertebral disc cell senescence and ECM catabolism in human intervertebral disc disease.^24^, ^25^ PI3K signalling has been widely reported to play a crucial role in the pathogenesis of atherosclerosis, thrombosis and myocardial infarction, but not in valvulopathies.^26^ Considering the importance of this pathway in a range of degenerative diseases, the data from our own preliminary studies and the lack of data on PI3K/AKT/mTOR signalling pathway in the development of MMVD, we believe investigating this pathway would be beneficial.

Examining the pathogenesis of MMVD in dogs will have relevance to understanding the same disease in humans. Studying the pathogenesis of human MMVD has relied on the use of transgenic mouse models and examining surgically‐resected mitral valve samples from patients with end‐stage disease. Although genetically modified mice give useful insights into many molecular signalling events, they are limited in modelling the triple layer structure of human valves and the chronicity of this disease and are not able to generate mitral‐valve specific myxomatous pathology.^27^, ^28^, ^29^, ^30^ Furthermore, human patient‐derived tissues (end‐stage disease) typically have extensive secondary fibrosis that hampers the examination of molecular events controlling the much earlier development and progression of the pre‐fibrosis myxomatous changes.^11^, ^12^, ^31^ The dog with the same triple‐layer valvular structure, but lacking the end‐stage fibrosis, has shared pathological and molecular characteristics of human MMVD and can be examined as the disease appears and progresses. This naturally occurring analogous disease in dogs is now well recognized as a credible large animal model to investigate human MMVD.^1^, ^3^, ^14^ By examining cell and molecular events in the dog, we can gain insights into MMVD in both species.

To that end, in the present study, we have performed in vitro mechanistic studies on cultured VICs isolated from healthy dogs and dogs with spontaneously developed mid‐stage MMVD to examine the role of PI3K/AKT/mTOR signalling in MMVD.

## 2 MATERIALS AND METHODS

### 2.1 ETHICS STATEMENT

All tissue collection procedures were performed under the approval and guidance of the Veterinary Ethics Research Committee (Institutional Care and Use Committee; project number 96/21) at The Royal (Dick) School of Veterinary Studies, University of Edinburgh. Written informed consent was obtained from each dog owner and no dogs were euthanized for the purpose of this study.

### 2.2 Clinical samples

Six mitral valves from diseased dogs of various breeds with MMVD and six mitral valves from healthy young adult dogs of various breeds were collected at the Hospital for Small Animals, The Royal (Dick) School of Veterinary Studies, University of Edinburgh. Collected resected valves were graded according to their gross pathological appearance normal (grade 0) or diseased (grades 1–4) using the Whitney classification, and graded independently by two observers.^32^ For this study, all six affected dogs were Whitney grade 2 (moderate disease).

### 2.3 Cell isolation, culture and phenotyping

Diseased canine VICs were isolated from the whole valves from dogs with grade 2 MMVD (moderately affected), and healthy VICs were isolated similarly from healthy dogs' valves. Briefly, canine mitral valve leaflets were rapidly removed, dissected, phenotyped and prepared for cell culture as previously described.^18^ Dissected valves were then incubated with 1 mg/mL trypsin (Gibco) for 10 min and washed in HBSS buffer (Gibco) to remove valve endothelial cells.^33^ The valve tissues were then digested in 250 U/mL type II collagenase solution (Worthington) at 37°C for 18 h. The cells subsequently obtained were resuspended in a low‐serum DMEM medium (Gibco) supplemented with 2% fetal bovine serum (FBS), 100 U/mL of penicillin and 100 mg/mL streptomycin (Gibco).^34^ Cells were cultivated using standard tissue culture techniques and used between 3 and 5 passages to ensure in vitro cultures maintain in vivo phenotype.^10^, ^11^ Cell phenotypes were determined by protein‐immunoblotting (Western blotting; WB) and quantitative PCR for the myofibroblast markers α‐SMA (*ACTA2*),^10^, ^11^ SM‐22 (*TAGLN*)^35^ and Smemb (*MYH10*).^18^, ^36^ All disease cell samples were positive for these markers and all normal cell samples were negative, confirming the accurate phenotype of the two groups.

### 2.4 Cell viability assay

Cell viability was measured with a commercial alamarBlue assay (Invitrogen). Briefly, cells were plated in a 96‐well plate for 24 h and then treated with a test compound before proceeding with the assay. The alamarBlue reagent was added directly to each well and incubated at 37°C for 3 h to allow cells to convert resazurin to resorufin. The absorbance at 570 nm for each well was measured using a microplate reader. The average 600 nm absorbance values of the background control were subtracted from the 570 nm absorbance values of experimental wells. The results were evaluated as background subtracted 570 nm absorbance versus concentration of the compounds.

### 2.5 siRNA transfection

aVICs were seeded at the density of 1.0 × 10^6^ cells/well in six‐well plates and transfected with 1.0 μM mouse p70 S6K siRNA (Santa Cruz Biotechnology), human p70 S6K siRNA (Santa Cruz Biotechnology), or scrambled control siRNA (Santa Cruz Biotechnology) using Lipofectamine 3000 (Invitrogen) in Opti‐MEM (Gibco) medium according to the manufacturer's instructions. aVICs transfected with Lipofectamine 3000 without p70 S6K siRNA were used as a mock control. aVICs with the expected density were treated with 10 ng/mL TGF‐β1 for 3 days to ensure the completion of senescent myofibroblast transition. These cells were then transfected with siRNAs. The siRNA sequences for gene silencing are listed in Table S1. RPS6KB1 genes responsible for the translation of p70 S6K protein is highly evolutionarily conserved in mouse, human and canine and therefore these siRNAs were used in this study.

### 2.6 Gene overexpression

Extraction of p70 S6K cDNA plasmids was performed with a Plasmid Plus Kit (QIAGEN) according to the manufacturer's instructions. qVICs were seeded into six‐well plates with a density of 1 × 10^6^ per well and cultured overnight. About 10 μg p70 S6K cDNA ORF plasmid (Genescript) was incubated with 50 μg Lipofectamine 3000 and 500 μL Opti‐MEM medium and then DNA‐Lipofectamine 3000 complexes were transferred to each well. Cells transfected with pcDNA3.1‐C‐(k) DYK vectors (Genescript) without p70 S6K cDNA were used as a negative control and cells transfected with Lipofectamine 3000 served as mock controls. The p70 S6K cDNA ORF clone sequences for gene overexpression are summarized in Table S2. Human and mouse p70 S6K cDNA ORF were used in this study because RPS6KB1 genes in these species share high homology.

### 2.7 Histology and immunohistochemistry

Canine mitral valve tissues were fixed with 10% (v/v) neutral buffer formalin (NBF) for 24 h, dehydrated and embedded in paraffin wax before sectioning at 3–5 μm using standard procedures. For evaluation of valve pathology, sections were dewaxed in xylene and ethanol and then stained with haematoxylin and eosin (H&E; Sangon Biotech). Light microscopy images were obtained by a scanning light microscope (Leica CS2) and histological analysis was performed. For immunohistochemistry, sections were subjected to sodium citrate buffer (pH 6.0) for antigen retrieval for 5 min at 95°C. Endogenous peroxidase activity was blocked using 1% hydrogen peroxide for 30 min at room temperature (RT). The blocking for non‐specific antibodies was performed with 10% normal goat serum (NGS) for 1 h at RT before overnight incubation at 4°C with rabbit anti‐PI3K 110α antibody (1:100, A94027, Antibodies), rabbit anti‐TGF‐β1 antibody (1:50, 21898‐1‐AP, Proteintech) and mouse anti‐α‐SMA antibody (1:300, #48938, Cell Signalling Technology) or mouse anti‐p21^CIP1^ antibody (1:100, 67362‐1‐Ig, Proteintech), mouse anti‐ATG7 antibody (1:100, 67341‐1‐Ig, Proteintech) and rabbit anti‐α‐SMA antibody (1:300, #19245, Cell Signalling Technology). PI3K 110α was selected as it is widely distributed in multiple tissues and p110 is the key catalytic subunit of the PI3K enzyme to trigger the downstream ATK/mTOR pathway.^37^ After washing in PBS slides were treated with Alexa Fluor 488 anti‐rabbit (1:500, Life Technologies), Alexa Fluor 645 anti‐mouse antibody (1:500, Life Technologies) or polymerized horseradish peroxidase (HRP) conjugated goat anti‐rabbit/mice antibody (1:1000, P0047/P0048, Dako) for 1 h at RT. Slides were then washed with PBS and finally stained with DAPI (1:5000, D9542, Sigma‐Aldrich) or 3,3′‐diaminobenzidine (DAB, SK‐4100, Vector Labs) followed by haematoxylin counterstain (H‐3401‐500, Vector Labs). Glass coverslips were mounted onto slides with Prolong Gold Anti‐Fade Reagent (Life Technologies). Control sections were incubated with equal concentrations of normal rabbit (ab172730, Abcam) and mouse IgG (ab37355, Abcam) in place of the primary antibody. The images were detected under an inverted confocal microscope (Zeiss LSM 710). Mean fluorescence intensity (MFI) and co‐localization analysis of TGF‐β1, PI3K 110α and α‐SMA were processed using ImageJ analysis software (National Institutes of Health).

### 2.8 Immunofluorescence staining

Cells seeded on glass coverslips were fixed with 10% (v/v) NBF at 4°C for 10 min, permeabilized in 0.1% (v/v) triton X‐100 (Sigma‐Aldrich) for 15 min and washed with PBS, followed by blocking in 5% NGS for 1 h at RT. Glass coverslips were washed in PBS and then incubated with rabbit anti‐LC3 antibody (1:300, PM036, MBL) at 4°C overnight. After washing, the cells were incubated with Alexa Fluor 488 anti‐rabbit antibody (1:500, A11034, Life Technologies) in NGS at 37°C for 1 h in the dark. Coverslips were then stained with Hoechst (1:10,000, 62249, Sigma‐Aldrich) and fluorescence signal was detected under an inverted confocal microscope (Zeiss LSM 710). Negative controls were carried out simultaneously by incubating with equivalent concentrations of normal rabbit IgG (ab172730, Abcam) in place of primary antibody.

### 2.9 Western blotting

VICs were collected with radioimmunoprecipitation assay (RIPA) lysis buffer (Thermo Fisher Scientific) supplemented with Protease and Phosphatase Inhibitor Cocktail (Thermo Fisher Scientific) and total protein concentration was determined (Thermo Fisher Scientific). Immunoblotting was performed as previously described.^38^ Equal amounts of protein lysates were separated by sodium dodecyl sulphate polyacrylamide gel electrophoresis (SDS‐PAGE) and transferred to polyvinylidene difluoride (PVDF) membranes (Millipore). After blocking with 5% (v/v) skimmed milk in Phosphate Buffered Saline Tween‐20 (PBST), membranes were incubated overnight at 4 °C with primary antibodies (Table S3) diluted in 5% skimmed milk. Subsequently, membranes were incubated with HRP‐conjugated anti‐mouse (1:1000, P0047, Dako) or anti‐rabbit (1:1000, P0048, Dako) secondary antibodies at RT for 1 h. Membranes were developed using the GeneGenome system (Syngene). Semi‐quantitative assessment of band intensity was performed using ImageJ analysis software (National Institutes of Health).

### 2.10 Terminal dUTP nick‐end labelling (TUNEL) staining

Apoptotic activities of canine VICs were identified using a fluorescein‐labelled TUNEL assay kit (ab252888, Abcam) following the manufacturer's instructions. In brief, coverslip‐seeded VICs were cultured with 60 μM LY294002 (Cayman Chemical), 5 μM copanlisib (Cayman Chemical), 50 μM alpelisib (Cayman Chemical) and DMSO (Sigma‐Aldrich) vehicle control for 3 days. Cells were then fixed and permeabilized followed by incubating with the TUNEL reaction cocktail overnight at RT. After washing in PBS, coverslips were treated with the click reaction cocktail and incubated for 30 min at RT in the dark. Cells were finally analysed for red fluorescence generated by TUNEL‐positive cells and green fluorescence by total DNA using an inverted confocal microscope (Zeiss LSM 710).

### 2.11 Flow cytometry

VICs treated with PI3K inhibitors and DMSO vehicle was harvested by trypsinization, washed with PBS and detached using trypsin. Cells are then resuspended with medium and counted using trypan blue to ensure dead cells are excluded. Cells were then stained with TUNEL reaction cocktail and the click reaction cocktail using TUNEL assay kit (ab252888, Abcam). Cell suspensions were finally transferred into flow cytometry vessels and 10,000 cell events were recorded in FL‐2 channel using a BD FACS Calibur Flow Cytometer (Becton, Dickinson & Company) for signals generated by TUNEL positive cells during click reaction.

### 2.12 BrdU cell proliferation assay

Cell proliferation was assessed using the BrdU Cell Proliferation ELISA Kit (Ab126556, Abcam) following the manufacturer's instructions. Briefly, 6 × 10^3^ cells were plated in a 96‐well plate and BrdU was added to the cells for 3 h. Subsequently, cells were subjected to fixation, permeabilization and DNA denaturation. Cells were then incubated with anti‐BrdU antibody for 1 h, washed and incubated with peroxidase‐conjugated secondary antibody. Finally, the coloured reaction that indicates cell proliferation was quantified at a wavelength of 450 nm.

### 2.13 Cell cycle analysis

For cell cycle determination, 4 × 10^5^ cells were rinsed with PBS and harvested by trypsinization. Pelleted cells were resuspended in 1 mL Hoechst‐solution containing 2 μg/mL Hoechst 33342 (H3570, Invitrogen) and incubated for 30 min. Subsequently, 1 μL 1 mg/mL 7‐aminoactinomycin D (7‐AAD) was added to the tube and incubated for 5 min to exclude apoptotic and dead cells.^39^ Fluorescent intact single nuclei were analysed for DNA content using cell analyser BD FACS Calibur Flow Cytometer (Becton, Dickinson & Company). Cell cycle was assessed through analysis of the proportion of cells in the G1, S and G2/M fraction of the cell cycle using FlowJo v10.8.

### 2.14 Senescence‐associated β‐galactosidase staining

Senescence‐associated β‐galactosidase (SA‐β‐gal) staining was performed according to the manufacturers' instructions (Merk Millipore). Briefly, canine VICs seeded in 12‐well plates were fixed in 0.25% glutaraldehyde and SA‐β‐gal staining was performed at pH 6.0. The percentage of SA‐β‐gal‐positive cells was quantified relative to the number of total cells, which were both counted in six random low‐power fields (×100) using the image analysis software ImageJ.

### 2.15 Quantitative real‐time PCR

Total RNAs were extracted from canine VICs using an RNeasy Kit (Qiagen) according to the manufacturer's instructions. RNA was quantified and reverse transcripted and the target gene expressions were evaluated by quantitative RT‐PCR in a 700 Fast Real‐Time PCR Systems (ViiA7 Real‐time PCR, ABI) using the SYBR™ Green PCR Master Mix (Thermo Fisher Scientific). Each PCR was run in triplicate. The relative expression levels of mRNAs were determined by a comparative 2^−Ct (ΔΔCt)^ method and normalized against glyceraldehyde‐3‐phosphate dehydrogenase (GAPDH). The control values were expressed as 1 to indicate a precise fold change value for each gene of interest. The primers used in this study were synthesized by Sigma‐Aldrich and the sequences for target genes are shown in Table S4.

### 2.16 Enzyme‐linked immunosorbent assay

The supernatants were collected from canine VIC cultures and centrifuged at 16,000 rpm at 4°C for 20 min to remove debris. The purified supernatants were diluted accordingly and examined by enzyme‐linked immunosorbent assays (ELISAs) using human TGF‐β1, interleukin‐6 (IL‐6) and matrix metalloproteinase‐9 (MMP‐9) ELISA kits (Invitrogen) according to the manufacturer's instructions. The genes responsible for the protein translation of TGF‐β1, IL‐6 and MMP‐9 are conserved in human and canine species.

### 2.17 Statistical analysis

All experiments were performed in three technical replicates with six biological replicates and the representative results are shown. All data are presented as mean ± SEM. Statistical analyses were analysed by one‐way analysis of variance (ANOVA) followed by Tukey's range test using GraphPad Prism software. *p* < 0.05 was considered to be significant, and *p* values are represented as: *p < 0.05; **p < 0.01; ***p < 0.001.

## 3 RESULTS

### 3.1 Expression of TGF‐β1 and PI3K 110α are significantly increased in α‐SMA positive aVICs in canine myxomatous mitral valves

**FIGURE 1:**
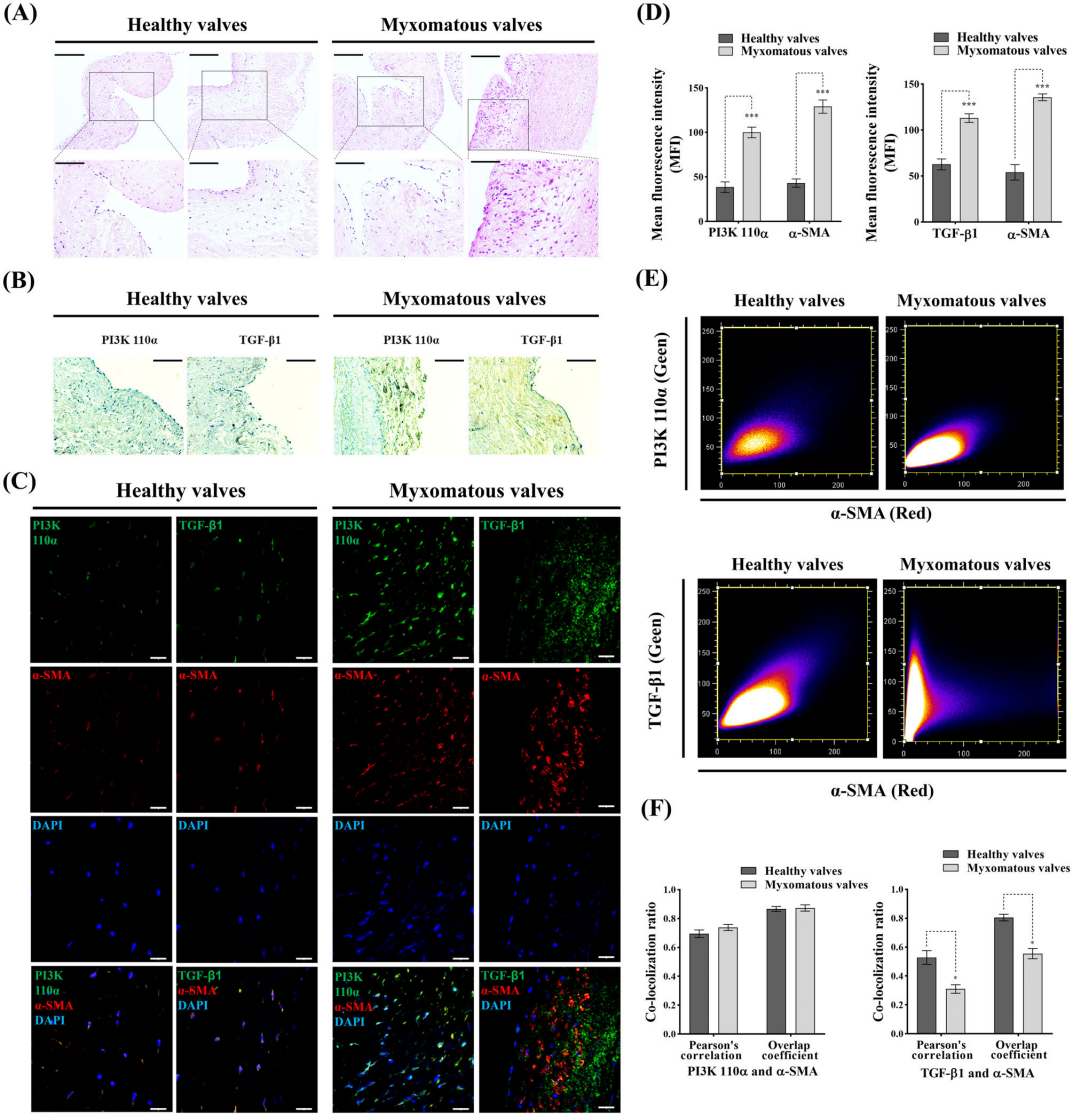
Histopathological assessment of canine healthy and myxomatous mitral valves. (A) Representative images (two biological replicates) of H&E staining in canine healthy and myxomatous mitral valves, scale bar 200 μm (magnified 100 μm). (B) Representative immunohistochemistry images of TGF‐β1 and PI3K 110α expressions (yellow/brown) in canine healthy and myxomatous mitral valves, scale bar 100 μm. (C) Representative confocal immunofluorescent images of TGF‐β1, PI3K 110α and α‐SMA expressions in canine healthy and myxomatous mitral valves, scale bar 20 μm. (D) Quantitative analysis of mean fluorescence intensity (MFI) of TGF‐β1, PI3K 110α and α‐SMA in canine healthy and myxomatous mitral valves. (E) Representative images of co‐localization ratio analysis of TGF‐β1 (green) and α‐SMA (red), PI3K 110α (green) and α‐SMA (red) fluorescence signals in canine mitral valve tissues. (F) Quantitative analysis of co‐localization parameters (Pearson's correlation and overlap coefficient) of TGF‐β1 (green) and α‐SMA (red), PI3K 110α (green) and α‐SMA (red) fluorescence signals. Results are presented as mean ± SEM. ANOVA followed by Tukey's range test. *p < 0.05, **p < 0.01, ***p < 0.01 compared to control. ANOVA, analysis of variance; H&E, haematoxylin and eosin.

To investigate the role of TGF‐β and PI3K in MMVD expression of TGF‐β1 and PI3K 110α was assessed in six canine healthy and six myxomatous mitral valves using IHC. Healthy valves were characterized by the normal triple‐layer valvular structure with no evidence of myxomatous lesions. Valves from dogs diagnosed with grade 2 MMVD had moderate myxomatous degeneration and no fibrotic changes (Figure 1A). Immunohistochemical assessment revealed TGF‐β1 and PI3K 110α expression within areas of ECM disorganization in myxomatous mitral tissues (Figure 1B). Double‐staining confocal immunofluorescence showed that TGF‐β1 and PI3K 110α expression were significantly increased in myxomatous valves, with a high density of α‐SMA+ cells, compared to healthy valves (Figure 1C,D).

To further confirm TGF‐β1 and PI3K 110α are expressed in α‐SMA positive aVICs, co‐localization analysis of PI3K 110α, TGF‐β1 and α‐SMA was performed. There was a high level of PI3K 110α and TGF‐β1 co‐localization with α‐SMA, but not between TGF‐β1 and α‐SMA in myxomatous valves (Figure 1E,F). These data indicate aVICs highly express PI3K 110α and TGF‐β1, and TGF‐β1 was secreted into the valvular matrix in myxomatous degeneration.

### 3.2 PI3K/AKT/mTOR/p70 S6K signalling is upregulated in α‐SMA positive aVICs

**FIGURE 2:**
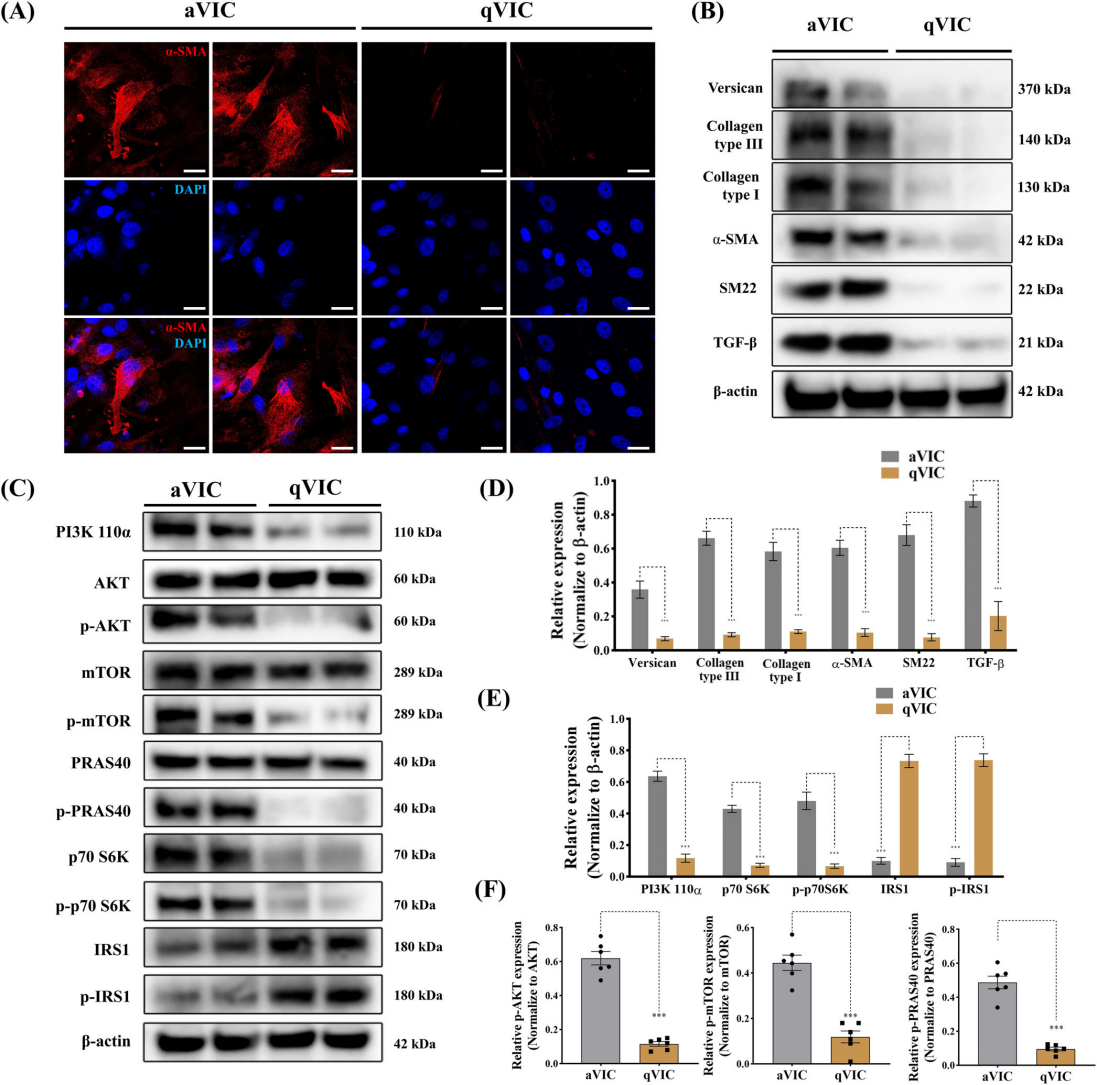
PI3K/AKT/mTOR/p70 S6K signalling is upregulated in activated myofibroblasts (aVICs). (A) Representative confocal images of α‐SMA (red) staining in canine aVICs and qVICs (two biological replicates), scale bar 20 μm. (B, D) Representative western blot of ECM and VIC phenotype protein expression and quantification of the relative protein expression (two biological replicates shown in blots, *n* = 6). (C, E, F) Representative western blot of PI3K 110α, total AKT, phosphorylated AKT (p‐AKT), total mTOR, phosphorylated mTOR (p‐mTOR), p70 S6K, phosphorylated p70 S6K (p‐p70 S6K), PRSA40, phosphorylated PRSA40 (p‐PRSA40), IRS1 and phosphorylated IRS1 (p‐IRS1) protein expression and quantification of the relative protein expression (two biological replicates shown in blots, *n* = 6). Results are presented as mean ± SEM. ANOVA followed by Tukey's range test. *p < 0.05, **p < 0.01, ***p < 0.001 compared to control. ANOVA, analysis of variance; ECM, extracellular matrix; VIC, valve interstitial cell.

Initially, studies were performed to validate the canine VIC in vitro 2D low‐serum culture model as previously reported.^18^ aVICs stained positive for the myofibroblast marker α‐SMA (Figure 2A). In addition, western blotting identified significantly increased expression of myofibroblast‐related cytoskeletal proteins including α‐SMA and SM22‐α (Figure 2B,D). The synthesis of the ECM proteins versican, collagen type I and collagen type III was significantly increased in aVICs, as was the expression of TGF‐β (Figure 2B,D). All these data are consistent with previous reports and confirm the phenotype of the normal and diseased samples.^16^, ^19^, ^40^ To investigate the mechanisms controlling VIC phenotype transition, we investigated the activation of PI3K/AKT/mTOR signalling. The baseline expression of PI3K signalling was evaluated by western blotting. PI3K 110α expression was significantly increased as were the phosphorylated forms of the downstream signalling molecules Akt Ser473, mTOR Ser2448 and the downstream mTOR transcriptional factor p70 S6 kinase (S6K) Thr389, which most closely correlates with its p70 kinase activity (Figure 2C,E,F).^41^ Considering the phosphorylation of p70 S6K can be controlled by insulin‐mediated insulin receptor (IR)/PI3K signalling, we assessed the baseline expression of insulin receptor substrate 1 (IRS‐1), the main substrate of IR kinase which activates PI3K/AKT/mTOR signalling.^42^ Protein expression of total IRS‐1 and its phosphorylated form at Ser636/639 were significantly reduced in aVICs (Figure 2C,E). Furthermore, proline‐rich AKT substrate of 40 kDa (PRAS40) interacts with raptor in mTOR complex 1 (mTORC1) and inhibits the activation of the mTORC1/S6K pathway, while phosphorylation of PRAS40 at Thr246 by AKT relieves this PRAS40 inhibition of mTORC1.^43^, ^44^ Interestingly we identified increased expression of phosphorylation of PRAS40 (Thr246) in aVICs but no significant difference in total PRAS40 expression comparing aVICs and qVICs (Figure 2C,F). These data indicate activation of PI3K signalling is associated with the abnormal VIC phenotype transition and ECM protein expression.

### 3.3 TGF‐β‐induced PI3K signalling activation regulates VIC phenotype differentiation and ECM protein synthesis

**FIGURE 3:**
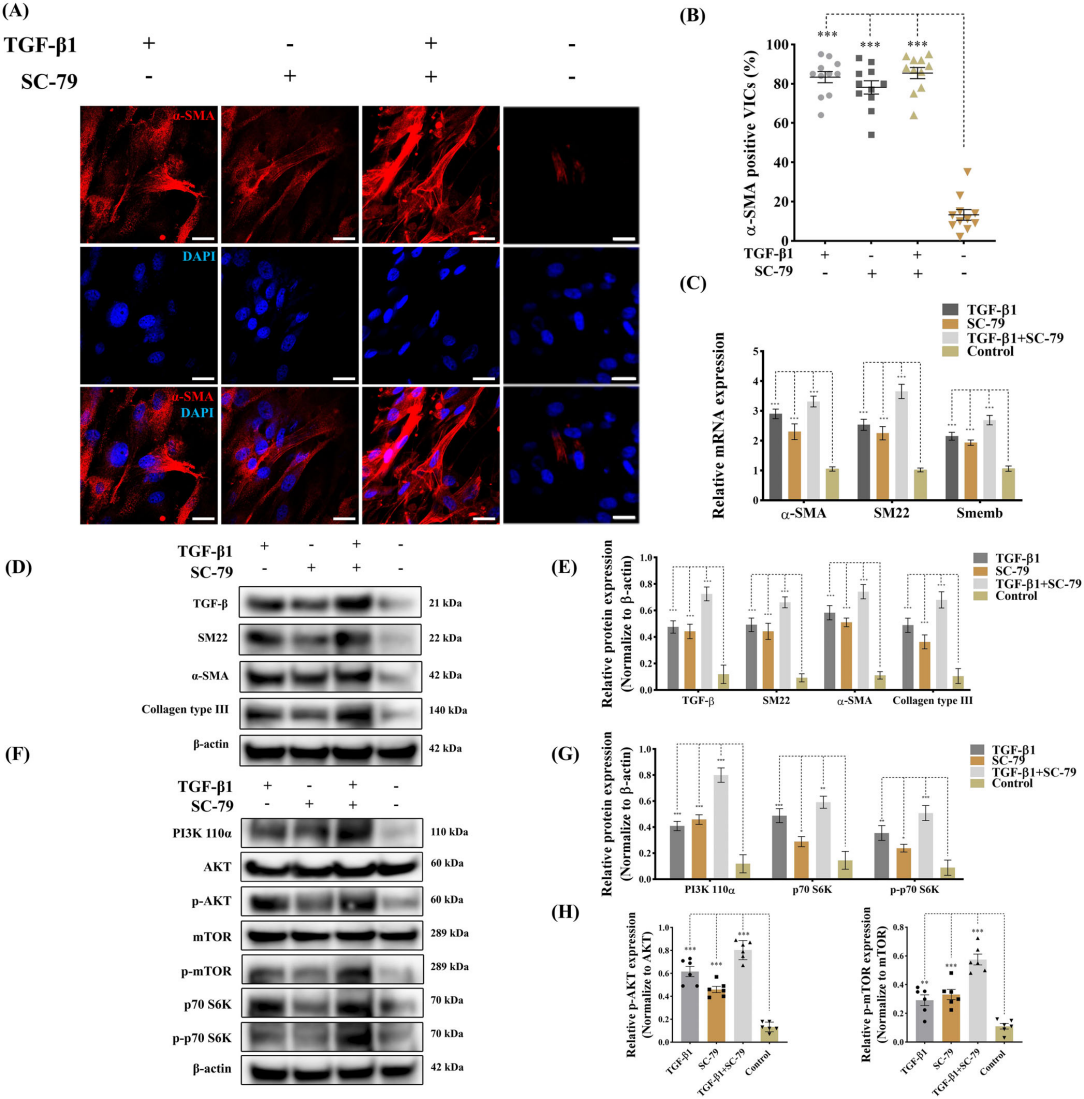
TGF‐β‐induced PI3K activation controls VIC phenotype and ECM protein production. Canine qVICs were exposed to DMSO (Control), TGF‐β1 (10 ng/mL) and SC‐79 (300 nM) treatment. (A, B) Representative confocal images of α‐SMA immunostaining and quantitative analysis of the percentage of α‐SMA positive cells treated with DMSO, TGF‐β1 (10 ng/mL) and SC‐79 (300 nM), scale bar 20 μm (*n* = 12 microscopic fields). (C) Quantitative RT‐PCR for α‐SMA, SM22 and Smemb mRNA expression in qVICs treated with DMSO, TGF‐β1 (10 ng/mL) and SC‐79 (300 nM) (*n* = 6). (D, E) Representative western blot of α‐SMA, SM22, collagen type III and TGF‐β and quantitative analysis of the relative protein expression (*n* = 6). (F, G, H) Representative western blot of PI3K p110α, AKT, phosphorylated AKT (p‐AKT), mTOR, phosphorylated mTOR (p‐mTOR), p70 S6K, phosphorylated p70 S6K (p‐p70 S6K) protein expression and quantitative analysis of the relative protein expression (*n* = 6). Results are presented as mean ± SEM. ANOVA followed by Tukey's range test. *p < 0.05, **p < 0.01, ***p < 0.001 compared to control. ANOVA, analysis of variance; ECM, extracellular matrix; TGF‐β, transforming growth factor β; VIC, valve interstitial cell.

To determine if TGF‐β mediated PI3K signalling controls myofibroblast activation qVICs were treated with TGF‐β1 (10 ng/mL) for 3 days and then exposed to the selective Akt activator SC‐79 (300 nM) for 2 h.^45^ SC‐79 is known to increase the cell's responsiveness to TGF‐β by inducing the transport of TGFBRs (TGF‐β receptors) to the cell surface.^46^, ^47^ The appropriate concentration of SC‐79 was determined by cell viability assays (Figure S1). α‐SMA immunostaining showed that TGF‐β1 in the presence or absence of SC‐79 treatment induced α‐SMA expression and largely increased α‐SMA positive VICs by Day 3 (Figure 3A,B). Myofibroblast‐related genes, including *ACTA2* (α‐SMA), *TAGLN* (SM22) and *MYH10* (Smemb), were dramatically upregulated after TGF‐β1 induction in qVICs cultured with low‐serum media supplemented with SC‐79 (Figure 3C). The protein expression of α‐SMA, SM22, collagen type III and TGF‐β in qVICs was significantly increased by TGF‐β1 treatment, again in both the presence or absence of SC‐79 (Figure 3D,E). In addition to the induction of canonical Smad‐mediated signalling, TGF‐β is known to initiate the non‐canonical PI3K/AKT/mTOR pathway.^48^ Western blotting revealed that TGF‐β1 treatment activated PI3K signalling by increasing PI3K 110α, phosphorylated AKT, phosphorylated mTOR, p70 S6K, phosphorylated p70 S6K expressions in qVICs (Figure 3F–H) in the presence or absence of SC‐79. These data show that TGF‐β‐induced PI3K signalling activation results in an aberrant transformation of VIC phenotype and ECM protein synthesis.

### 3.4 aVICs exhibit a senescent‐associated secretory phenotype with a reduced capacity for autophagy

**FIGURE 4:**
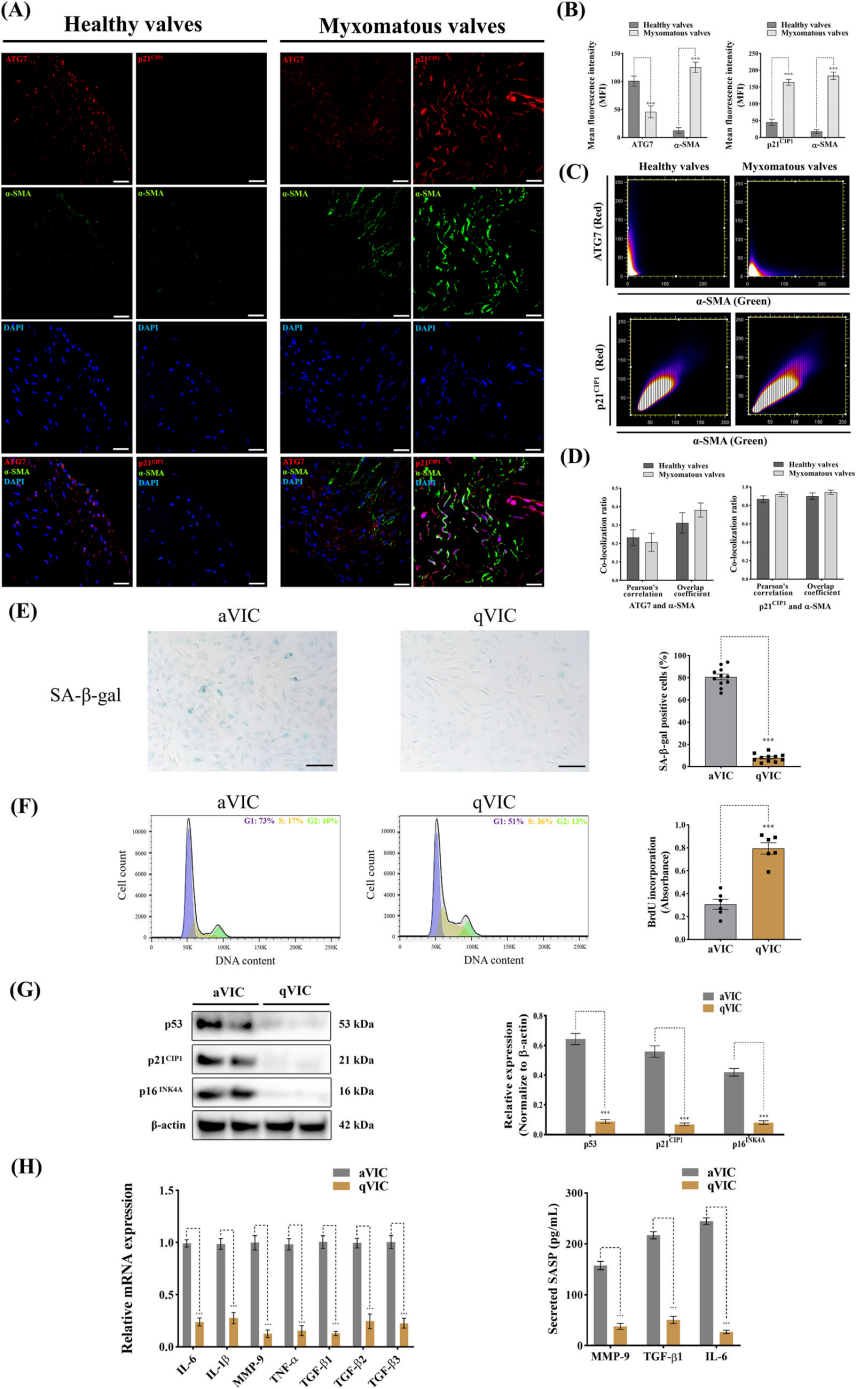
Activated myofibroblasts (aVICs) exhibit a senescent‐associated secretory phenotype (SASP) with a reduced capacity for autophagy. (A) Representative confocal immunofluorescent images of ATG7, p21^CIP1^ and α‐SMA expressions in canine healthy and myxomatous mitral valves, scale bar 20 μm. (B) Quantitative analysis of mean fluorescence intensity (MFI) of ATG7, p21^CIP1^ and α‐SMA in canine healthy and myxomatous mitral valves. (C) Representative images of co‐localization ratio analysis of ATG7 (red) and α‐SMA (green), p21^CIP1^ (red) and α‐SMA (green) fluorescence signals in canine mitral valve tissues. (D) Quantitative analysis of co‐localization parameters (Pearson's correlation and overlap coefficient) of ATG7 (red) and α‐SMA (green), p21^CIP1^ (red) and α‐SMA (green) fluorescence signals. (E) Representative images of SA‐β‐gal (blue) staining and quantitative analysis of the percentage of SA‐β‐gal positive cells, scale bar 50 μm (*n* = 12 microscopic fields/treatment). (F) Cell cycle analysis (left panel) and BrdU incorporation assay (right panel) of canine aVICs and qVICs (*n* = 6). (G) Representative western blot of p16^INK4A^, p21^CIP1^, p53 and β‐actin protein expression and quantification of the relative protein expression in VICs (two biological replicates shown in blots, *n* = 6). (H) Quantitative RT‐PCR for SASP cytokine expression (left panel) and secreted TGF‐β1, IL‐6 and MMP‐9 (right panel) in collected supernatant from VIC cultures (*n* = 6). Results are presented as mean ± SEM. ANOVA followed by Tukey's range test. *p < 0.05, **p < 0.01, ***p < 0.001 compared to control. ANOVA, analysis of variance; VIC, valve interstitial cell.

Cell senescence has been shown to be modulated by PI3K signalling.^49^, ^50^ Autophagy impairment is considered as an important characteristic of cell senescence.^51^ To determine whether aVICs are in a senescent phenotype, p21^CIP1^ and ATG7 expression profiles were examined in mitral valve tissues by double‐staining confocal immunofluorescence p21^CIP1^ expression was significantly increased while conversely ATG7 expression was significantly decreased in myxomatous valves compared to healthy valves (Figure 4A,B). To further confirm whether ATG7 and p21^CIP1^ are expressed in α‐SMA positive aVICs, co‐localization analysis of ATG7, p21^CIP1^ and α‐SMA was performed. There was a high level of p21^CIP1^ co‐localization with α‐SMA, but not between ATG7 and α‐SMA in myxomatous valves (Figure 4C,D). These data indicate aVICs are in a senescent state but with a reduced autophagy flux in myxomatous valves.

To further confirm aVICs are senescent, SA‐β‐gal staining was performed on canine in vitro VIC cell cultures. A larger number of SA‐β‐gal positive cells were observed in aVICs compared with qVICs (Figure 4E). Cell cycle analysis showed that a higher percentage of aVICs accumulated in the G1 phase, together with a reduced number of S and G2/M phase cells compared with qVICs (Figure 4F). In addition, cell proliferation was determined by the measurement of newly synthesized DNA using the thymidine analogue BrdU. qVICs exhibited a significantly increased capacity for BrdU incorporation compared to aVICs (Figure 4F). p53/p21^CIP1^ and p16^INK4A^ tumour suppressor signalling has been reported as the key pathways involved in the activation of cellular senescence.^52^ p16^INK4A^, p53 and p21^CIP1^ protein expressions were significantly increased in aVICs (Figure 4G). Taken together, these data indicate aVICs are in a senescent state.

Senescent cells can develop a senescence‐associated secretory phenotype (SASP), which allows them to secrete a complex mixture of factors causing continual ECM disorganization and alter the behaviour of nearby non‐senescent cells. The main components of SASP include multiple pro‐inflammatory cytokines, chemokines, growth modulators, ECM components, and MMPs.^52^ To examine SASP in aVICs, a series of SASP members, including IL‐6, IL‐1β, MMP‐9, TNF‐α, TGF‐β1, TGF‐β2 and TGF‐β3, were selected for quantitative RT‐PCR analysis. The upregulation of the selected SASP mRNAs was observed in aVICs (Figure 4H). Detected by ELISA TGF‐β1, IL‐6 and MMP‐9 levels in the culture supernatant from aVICs were significantly increased compared to qVICs (Figure 4H). Taken together, these data suggest that senescent aVICs exhibit a SASP.

### 3.5 Pharmacological inhibition of PI3K signalling reverses myofibroblast activation and normalized ECM production

**FIGURE 5:**
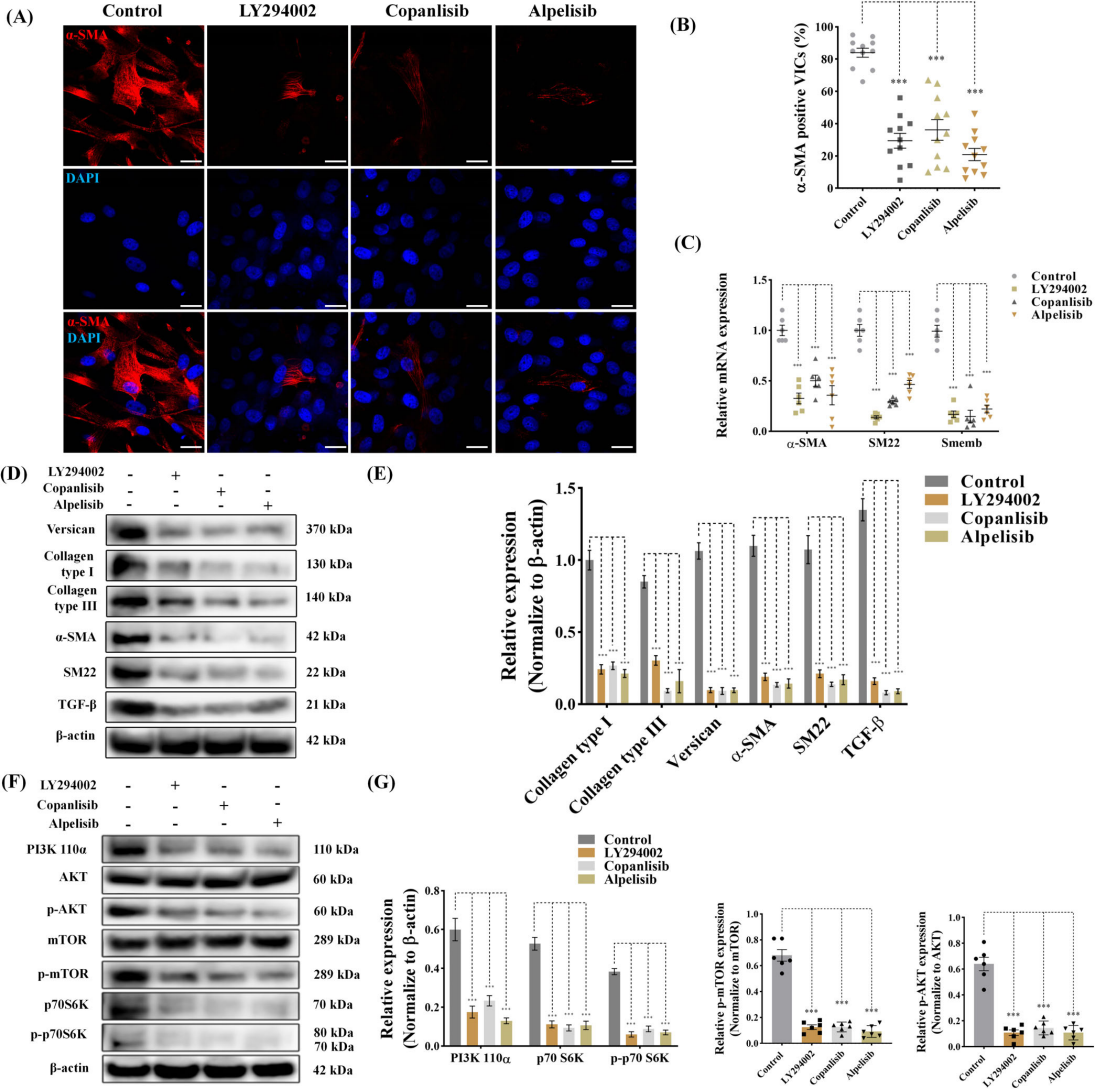
Pharmacological inhibition of PI3K signalling reverses VIC phenotype and reduces ECM protein expression in canine aVICs. aVICs were exposed to DMSO (Control), LY294002 (60 μM), copanlisib (5 μM) and alpelisib (50 μM) treatment for 3 days. (A, B) Representative confocal images of α‐SMA (red) staining in canine aVICs and quantitative analysis of the percentage of α‐SMA positive cells, scale bar 20 μm (*n* = 12 microscopic fields/treatment). (C) Quantitative RT‐PCR for α‐SMA, SM22 and Smemb mRNA expression in aVICs (*n* = 6) at Day 3. (D, E) Representative western blot of ECM and VIC phenotype protein expression and quantification of the relative protein expression (*n* = 6). (F, G) Representative western blot of PI3K 110α, AKT, phosphorylated AKT (p‐AKT), mTOR, phosphorylated mTOR (p‐mTOR), p70 S6K, phosphorylated p70 S6K (p‐p70 S6K) protein expression and quantification of the relative protein expression (*n* = 6). Results are presented as mean ± SEM. ANOVA followed by Tukey's range test. *p < 0.05, **p < 0.01, ***p < 0.001 compared to control. ANOVA, analysis of variance; ECM, extracellular matrix; VIC, valve interstitial cell.

To attenuate the aberrant activation of PI3K signalling in aVICs, we treated aVICs with 60 μM LY294002 (highly selective pan‐inhibitor of PI3K), 5 μM copanlisib (pan‐PI3K inhibitor) and 50 μM alpelisib (isoform‐selective PI3K p110α inhibitor) for 3 days.^37^ The optimal concentrations of the three PI3K inhibitors were determined by cell viability assays (Figure S1). LY294002, copanlisib and alpelisib attenuated α‐SMA expression and significantly reduced the number of α‐SMA positive VICs (Figures 4B and 5A,B). The upregulation of *ACTA2* (α‐SMA), *TAGLN* (SM22‐α) and *MYC10* (Smemb) mRNA expression in aVICs were significantly attenuated by LY294002 treatment (Figure 5C). Similar results were observed with copanlisib or alpelisib treatment (Figure 5C). Moreover, western blotting revealed that expression of α‐SMA, SM22‐α, TGF‐β and ECM protein collagen type I, collagen type III and versican was reduced by LY294002, copanlisib and alpelisib (Figure 5D,E). As expected, LY294002, copanlisib and alpelisib significantly reduced the expression of PI3K 110α, p70 S6K, phosphorylated p70 S6K, phosphorylated AKT and phosphorylated mTOR expression in aVICs (Figure 5F,G).

### 3.6 Antagonism of PI3K pathway promotes VIC apoptosis

**FIGURE 6:**
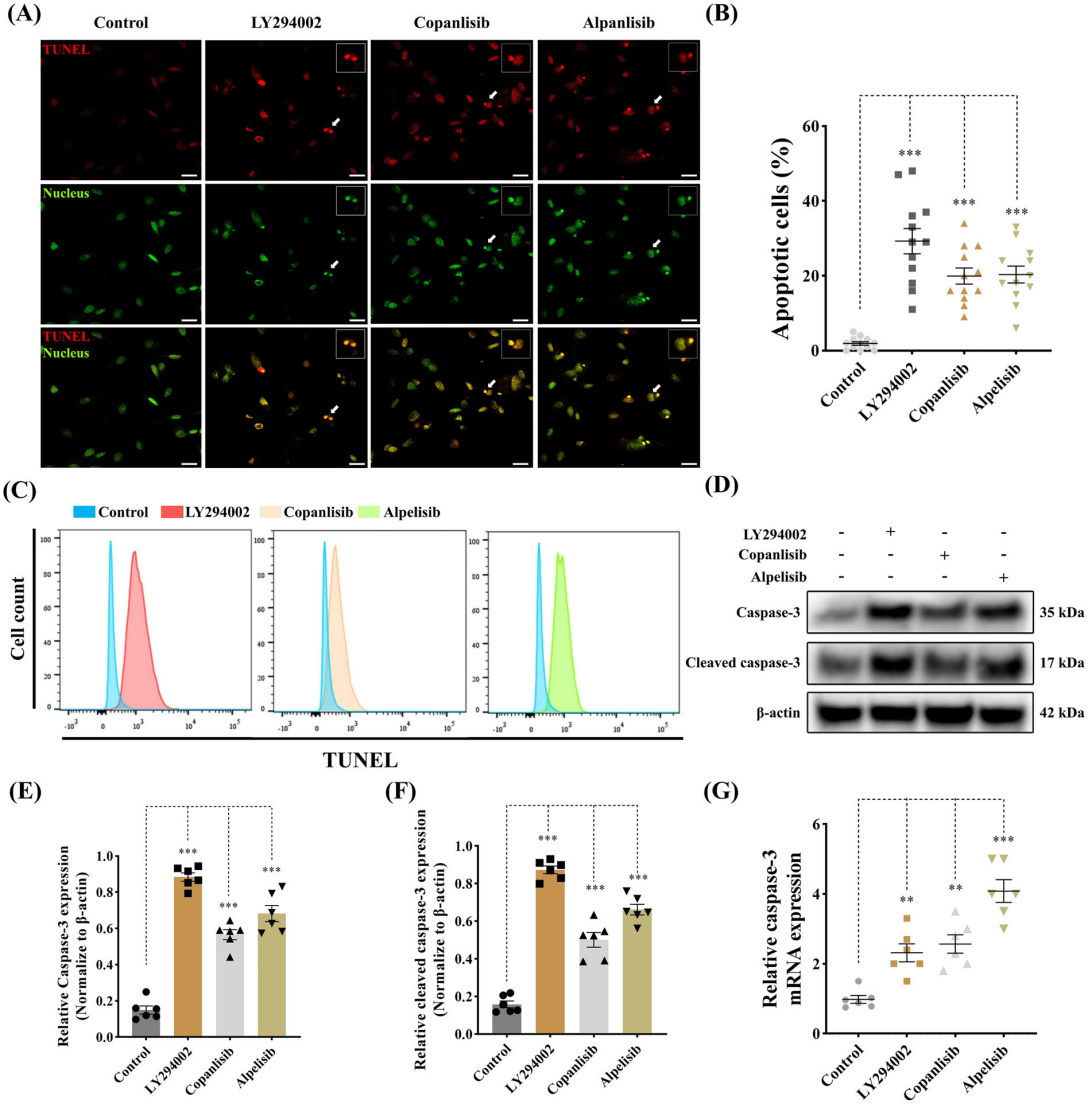
Antagonism of PI3K pathway promotes apoptosis in canine aVICs. aVICs were treated with DMSO (Control), LY294002 (60 μM), copanlisib (5 μM) and alpelisib (50 μM) for 3 days. (A, B) Representative confocal images of TUNEL (red) staining and quantitative analysis of the percentage of TUNEL positive (apoptotic) cells (arrowhead), scale bar 20 μm (*n* = 12 microscopic fields/treatment). (C) Flow cytometry analysis of TUNEL staining and quantification of the percentage of apoptotic cells at Day 3 (*n* = 3). (D–F) Representative western blot of caspase‐3, cleaved caspase‐3 and β‐Actin protein expression and quantification of the relative protein expression (*n* = 6). (G) Quantitative RT‐PCR for caspase‐3 mRNA expression in aVICs treated with PI3K inhibitors after 3 days (*n* = 6). Results are presented as mean ± SEM. ANOVA followed by Tukey's range test. *p < 0.05, **p < 0.01, ***p < 0.001 compared to control. ANOVA, analysis of variance; aVIC, activated myofibroblast phenotype.

PI3K signalling has been previously reported to play a crucial role in the regulation of cell proliferation and apoptosis.^21^ To determine if PI3K antagonism can affect apoptosis aVICs were treated with 60 μM LY294002, 5 μM copanlisib and 50 μM alpelisib for 3 days, as described earlier, followed by TUNEL staining and examination by confocal microscopy. The number of TUNEL‐positive (apoptotic) cells was markedly increased, with nuclear fragmentation, chromatin condensation, chromatin and apoptotic body formation observed (Figure 6A,B). Flow cytometry confirmed that the TUNEL‐positive cells were increased after treatment with one of the three antagonists (Figure 6C). Since caspase‐3 has been shown as the key terminal executioner of caspase‐activated both by extrinsic and intrinsic apoptosis pathways, Western blotting and quantitative PCR were performed to examine the expression of caspase‐3 and cleaved caspase‐3.^53^ Treatment by all three antagonists significantly increased the expression of caspase‐3 and cleaved caspase‐3 in aVICs (Figure 6D–G). Taken together, these data confirm pharmacological inhibition of the PI3K signalling pathway promotes aVIC cell apoptosis, and conversely that apoptosis is repressed in aVICs and in MMVD.

### 3.7 Suppression of PI3K signalling enhances VIC autophagy

**FIGURE 7:**
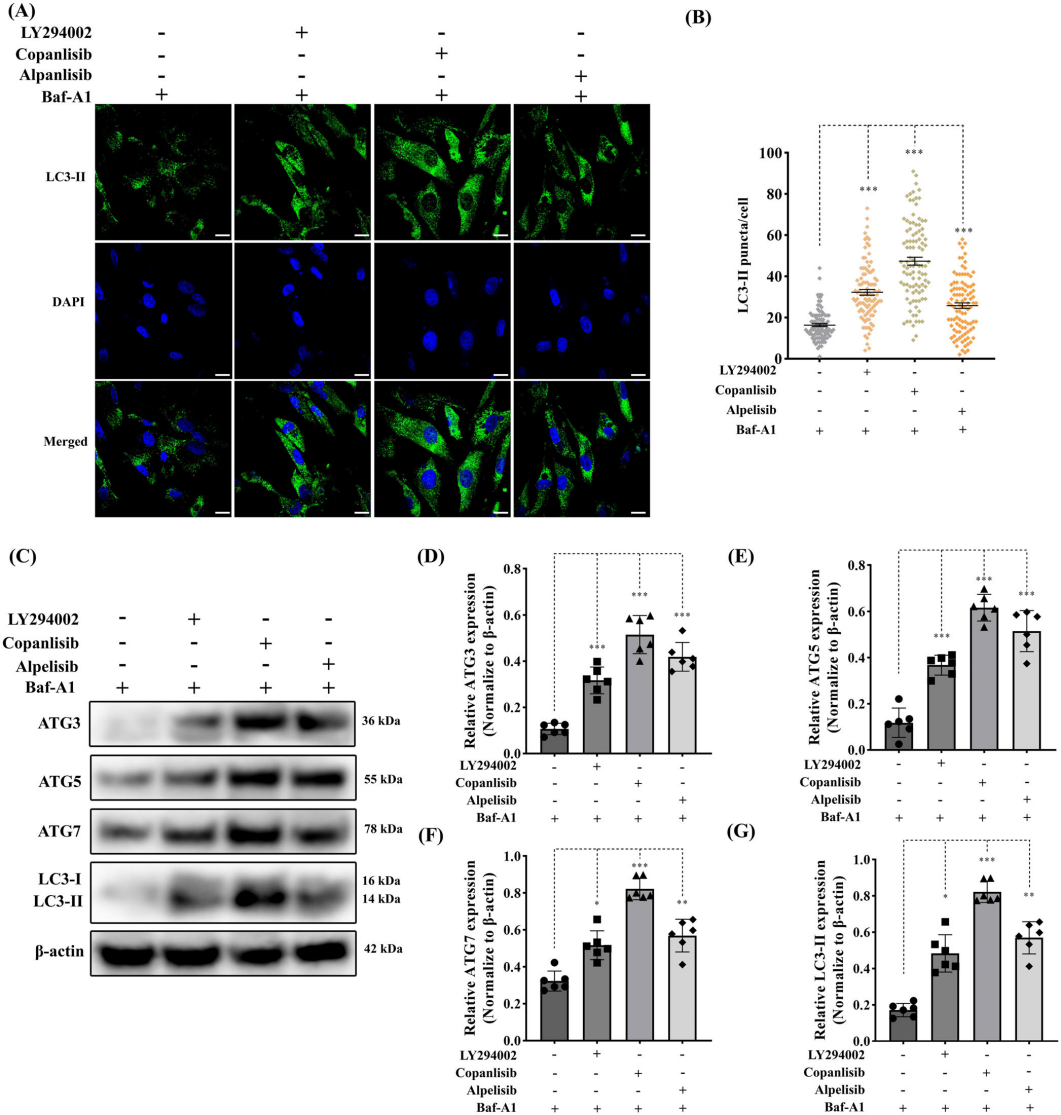
Inhibition of PI3K signalling enhances autophagy in canine aVICs. aVICs were exposed to DMSO (Control), 60 μM LY294002, 5 μM copanlisib and 50 μM alpelisib treatment with 5 μM baflomycin‐A1 (Baf‐A1) for 16 h. (A, B) Representative confocal images of LC3‐II marked autophagosomes (green) and quantitative analysis of the number of LC3‐II puncta, scale bar 20 μm (*n* = 98 cells/treatment). (C) Representative western blot of ATG3, ATG5, ATG7 and LC3‐II protein expression in aVICs exposed to DMSO and PI3K inhibitors. (D–G) Quantification of the relative protein expression of ATG3, ATG5, ATG7 and LC3‐II (*n* = 6). Results are presented as mean ± SEM. ANOVA followed by Tukey's range test. *p < 0.05, **p < 0.01, ***p < 0.001 compared to control. ANOVA, analysis of variance; aVIC, activated myofibroblast phenotype.

In addition to cell apoptosis, PI3K signalling plays a key role in the control of autophagy through the modulation of the downstream effects mTOR/p70 S6K.^54^ aVICs were pre‐treated with 60 μM LY294002, 5 μM copanlisib and 50 μM alpelisib for 24 h, and then treated with 5 μM baflomycin‐A1 for 16 h to inhibit autophagy flux by blocking autolysosomal degradation.^55^ The appropriate concentrations of baflomycin‐A1 were assessed in VICs by cell viability assay (Figure S1). LC3‐II immunostaining was performed to examine the formation of autophagosomes in aVIC cytoplasm. LY294002, copanlisib and alpelisib treatment induced LC3‐II labelled autophagosome formation and LC3‐II puncta numbers were significantly increased in PI3K suppressed aVICs (Figure 7A,B). The formation of autophagosomes from phagophores has been reported to require the participation of the evolutionarily conserved autophagy‐related (ATG) genes; we therefore examined the expression of ATG3, ATG5, ATG7 and LC3‐II by Western Blotting (Figure 7C). Inhibition of PI3K signalling by the three antagonists significantly increased the expression of ATG3, ATG5, ATG7 and LC3‐II (Figure 7D–G). These data indicate that pharmacological inhibition of the PI3K signalling pathway promotes aVIC cell autophagy, and that autophagy is suppressed in aVICs and in MMVD.

### 3.8 Pharmacological antagonism of PI3K signalling reverses VIC cellular senescence and secretory phenotype

To investigate whether PI3K signalling antagonism has an effect on cell senescence, senescence associated‐β‐galactosidase (SA‐β‐gal) staining was performed to detect the status of senescence in aVICs treated with LY294002, copanlisib and alpelisib for 24 h.

**FIGURE 8:**
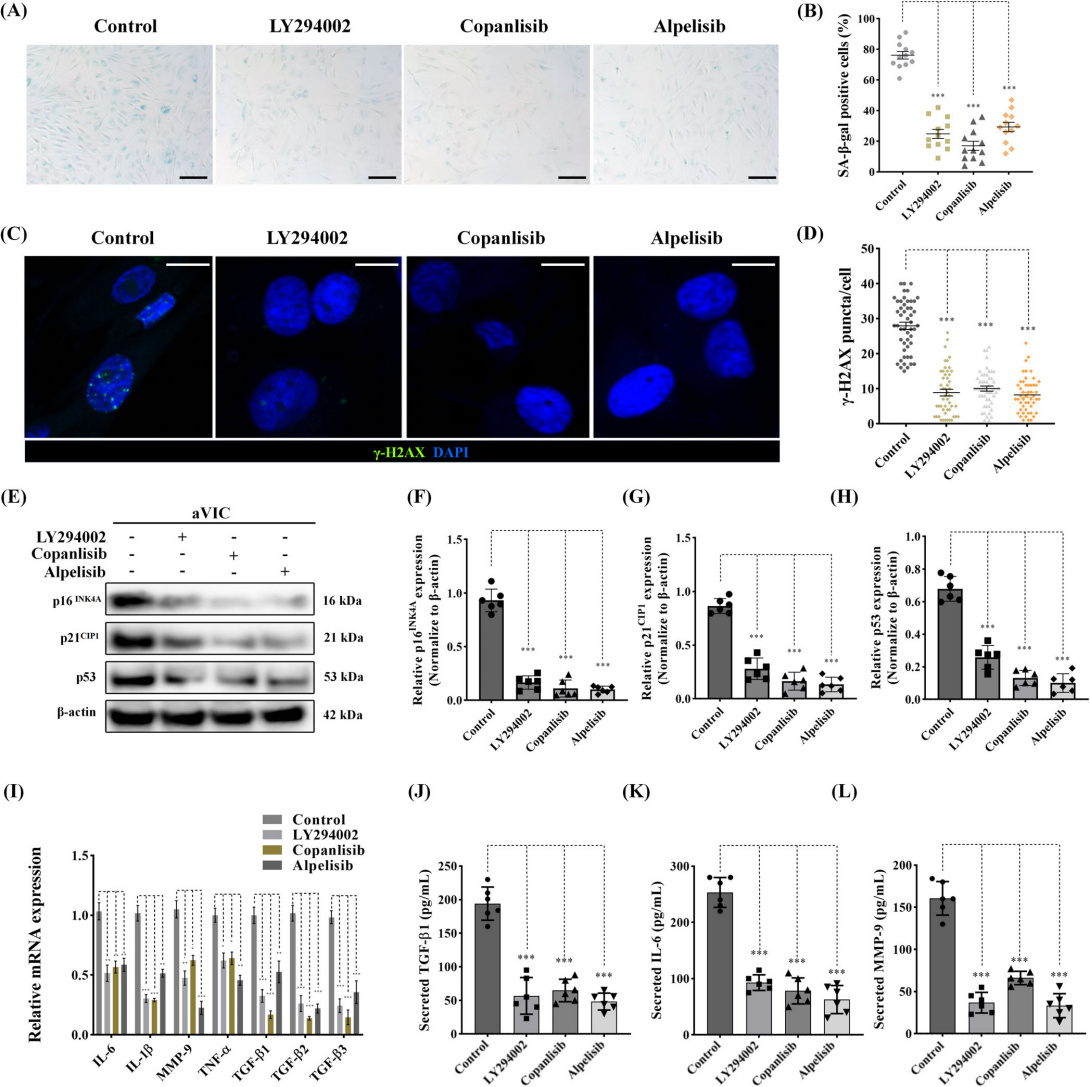
Pharmacological antagonism of PI3K signalling attenuates cellular senescence in canine aVICs. aVICs were exposed to DMSO (Control), 60 μM LY294002, 5 μM copanlisib and 50 μM alpelisib treatment for 24 h. (A, B) Representative images of SA‐β‐gal (blue) staining and quantitative analysis of the percentage of SA‐β‐gal positive cells, scale bar 50 μm (*n* = 12 microscopic fields/treatment). (C, D) Representative confocal images of γ‐H2AX (green) immunostaining in nuclei and quantitative analysis of the number of γ‐H2AX puncta, scale bar 20 μm (*n* = 50 cells/treatment). (E) Representative western blot of p16^INK4A^, p21^CIP1^, p53 and β‐actin protein expression and (F–H) quantification of the relative protein expression (*n* = 6). (I) Quantitative RT‐PCR for SASP cytokine expression in aVICs exposed to DMSO and PI3K inhibitors (*n* = 6). (J–L) Quantification of secreted TGF‐β1, IL‐6 and MMP‐9 in collected supernatant from aVIC cultures (*n* = 6). Results are presented as mean ± SEM. ANOVA followed by Tukey's range test. *p < 0.05, **p < 0.01, ***p < 0.001 compared to control. ANOVA, analysis of variance; aVIC, activated myofibroblast phenotype; SASP, senescent‐associated secretory phenotype.

There was a decrease in SA‐β‐gal positive staining after the inhibition of PI3K in aVICs (Figure 8A,B). Since DNA damage is also recognized as an important characteristic of senescent cells, we decided to examine the presence of discrete nuclear γ‐H2AX foci using immunofluorescence‐based confocal microscopy.^52^ The formation of γ‐H2AX foci was markedly reduced by LY294002, copanlisib and alpelisib treatment of aVICs (Figure 8C,D). p16^INK4A^, p53 and p21^CIP1^ protein expression were significantly decreased in treated aVICs (Figure 8E–H). However, the majority of treated cells accumulated in G1 phase, together with a decreased BrdU incorporation (Figure S2). The upregulation of the selected SASP mRNAs in aVICs was significantly attenuated by PI3K antagonism (Figure 8I). TGF‐β1, IL‐6 and MMP‐9 levels in the culture supernatant from aVICs were significantly reduced showed by ELISA (Figure 8J–L). Taken together, these data suggest that pharmacological inhibition of the PI3K signalling pathway abolishes cell senescence and SASP in aVICs returning cells to a more normal phenotype.

### 3.9 Upregulation of mTOR signalling by overexpressing p70 S6K induces activated myofibroblast differentiation and cellular senescence with a reduced capacity for apoptosis and autophagy

**FIGURE 9:**
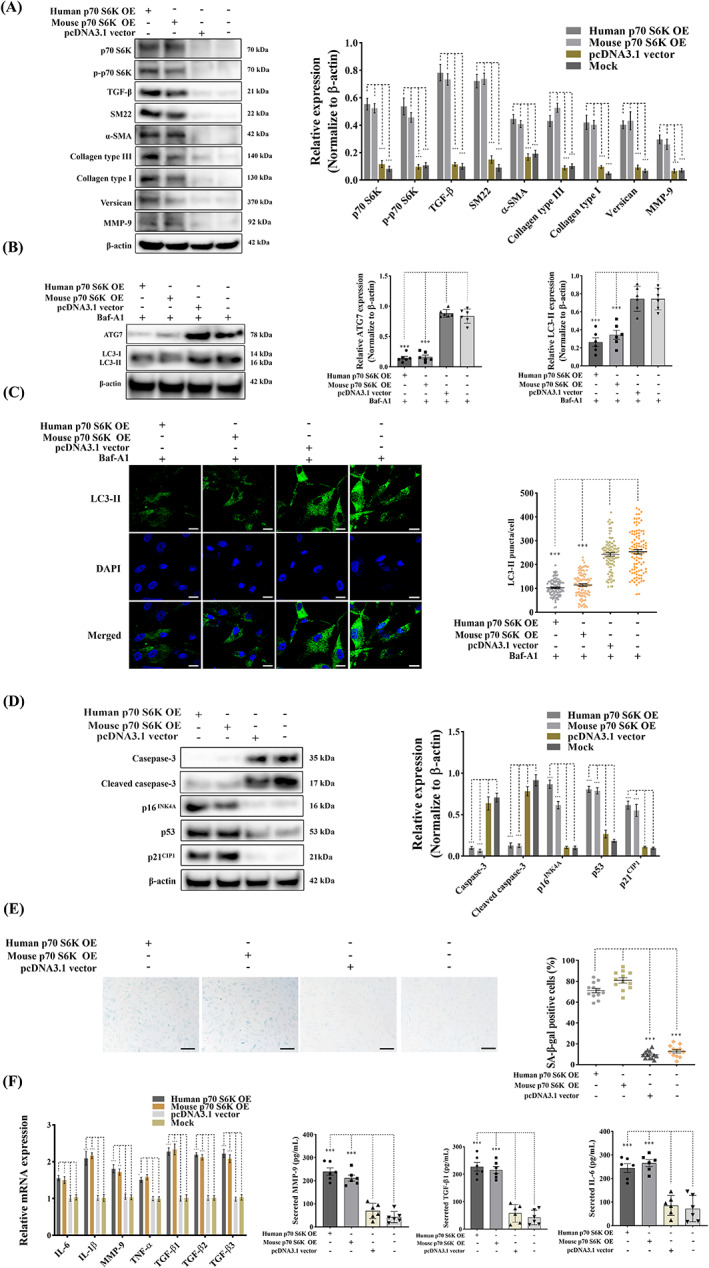
Overexpression of p70 S6K induces activated myofibroblast differentiation and ECM production through downregulating apoptosis and autophagy while enhancing cellular senescence. Canine qVICs were transfected with human p70 S6K cDNA ORF plasmids, mouse p70 S6K cDNA ORF plasmids, pcDNA3.1 plasmids (vectors) and Lipofectamine 3000 (Mock) with or without 5 μM of baflomycin‐A1 (Baf‐A1). (A) Representative western blot of p70 S6K, phosphorylated p70 S6K (p‐p70 S6K), VIC phenotype and ECM protein expression and quantification of the relative protein expression (*n* = 6). (B) p70 S6K overexpressed qVICs were exposed to 5 μM Baflomycin‐A1. Representative western blot of ATG7 and LC3‐II protein expression and quantification of the relative protein expression (*n* = 6). (C) Representative confocal images of LC3‐II labelled autophagosomes (green) and quantitative analysis of the number of LC3‐II puncta, scale bar 20 μm (*n* = 98 cells/treatment). (D) Representative western blot of caspase‐3, cleaved caspase‐3, p16^INK4A^, p21^CIP1^ and p53 protein expression and quantification of the relative protein expression (*n* = 6). (E) Representative images of SA‐β‐gal (blue) staining and quantitative analysis of the percentage of SA‐β‐gal positive cells, scale bar 50 μm (*n* = 12 microscopic fields). (F) Quantitative RT‐PCR for senescence‐associated secretory phenotype (SASP) cytokine expression (left panel) in qVICs transfected with p70 S6K cDNA ORF plasmids (*n* = 6). Quantification of secreted TGF‐β1, IL‐6 and MMP‐9 (right panel) in collected supernatant from qVIC cultures overexpressing p70 S6K (*n* = 6). Results are presented as mean ± SEM. ANOVA followed by Tukey's range test. *p < 0.05, **p < 0.01, ***p < 0.001 compared to control. ANOVA, analysis of variance; ECM, extracellular matrix; VIC, valve interstitial cell.

As a major downstream target of PI3K/AKT signalling mTOR occupies a pivotal position in the regulation of cell apoptosis, autophagy and senescence.^56^ Activation of the mTOR pathway initiates senescent p53/p21^CIP1^ signalling, inhibits caspases‐mediated apoptosis and inactivates ATG‐associated autophagy.^50^, ^57^ mTOR complex 1 (mTORC1) regulates these cellular activities at the transcriptional level by modulating the phosphorylation of the key downstream transcriptional factor ribosomal protein p70 S6K.^58^, ^59^ To elucidate the role of p70 S6K in aberrant qVIC myofibroblast differentiation and the modulation of these important cellular activities, human and mouse p70 S6K were overexpressed in canine qVICs separately using the DNA‐Lipofectamine method. Overexpression of p70 S6K resulted in the transition of qVICs to aVICs by inducing a significant increased protein expression of α‐SMA and SM22‐α. The synthesis of ECM proteins (collagen type I, collagen type III, versican), TGF‐β and MMP‐9 was markedly increased with VIC phenotype transformation (Figure 9A).

To clarify the status and role of autophagic flux p70 S6K overexpressed qVICs were treated with 5 μM baflomycin‐A1. The autophagic flux was compromised with the downregulation of ATG7 and LC3‐II expression (Figure 9B). LC3‐II immunostaining identified the LC3‐II puncta formation was largely inhibited by overexpression of p70 S6K (Figure 9C). p70 S6K overexpressed qVICs showed significantly increased expression of the senescent markers p16^INK4A^, p53 and p21^CIP1^ with a reduced caspase‐3 and cleaved caspase‐3 expression level (Figure 9D). Overexpression of p70 S6K also increased SA‐β‐gal positive cells (Figure 9E), significantly reduced BrdU incorporation (Figure S3B) and enhanced cells in G1 phase (Figure S3A), with concomitantly upregulated gene expression of SASP (genes for IL‐6, IL‐1β, MMP‐9, TNF‐α, TGF‐β1, TGF‐β2 and TGF‐β3) (Figure 9F). There was significantly increased expression of IL‐6, MMP‐9 and TGF‐β1 detected by ELISA (Figure 9F). These data show that overexpression of p70 S6K induces the activated myofibroblast differentiation, ECM disorganization and cellular senescence, while a reducing VIC capacity for apoptosis and autophagy.

### 3.10 Knockdown of p70 S6K revives VIC phenotype and alleviates cellular senescence with a promoted autophagic state

**FIGURE 10:**
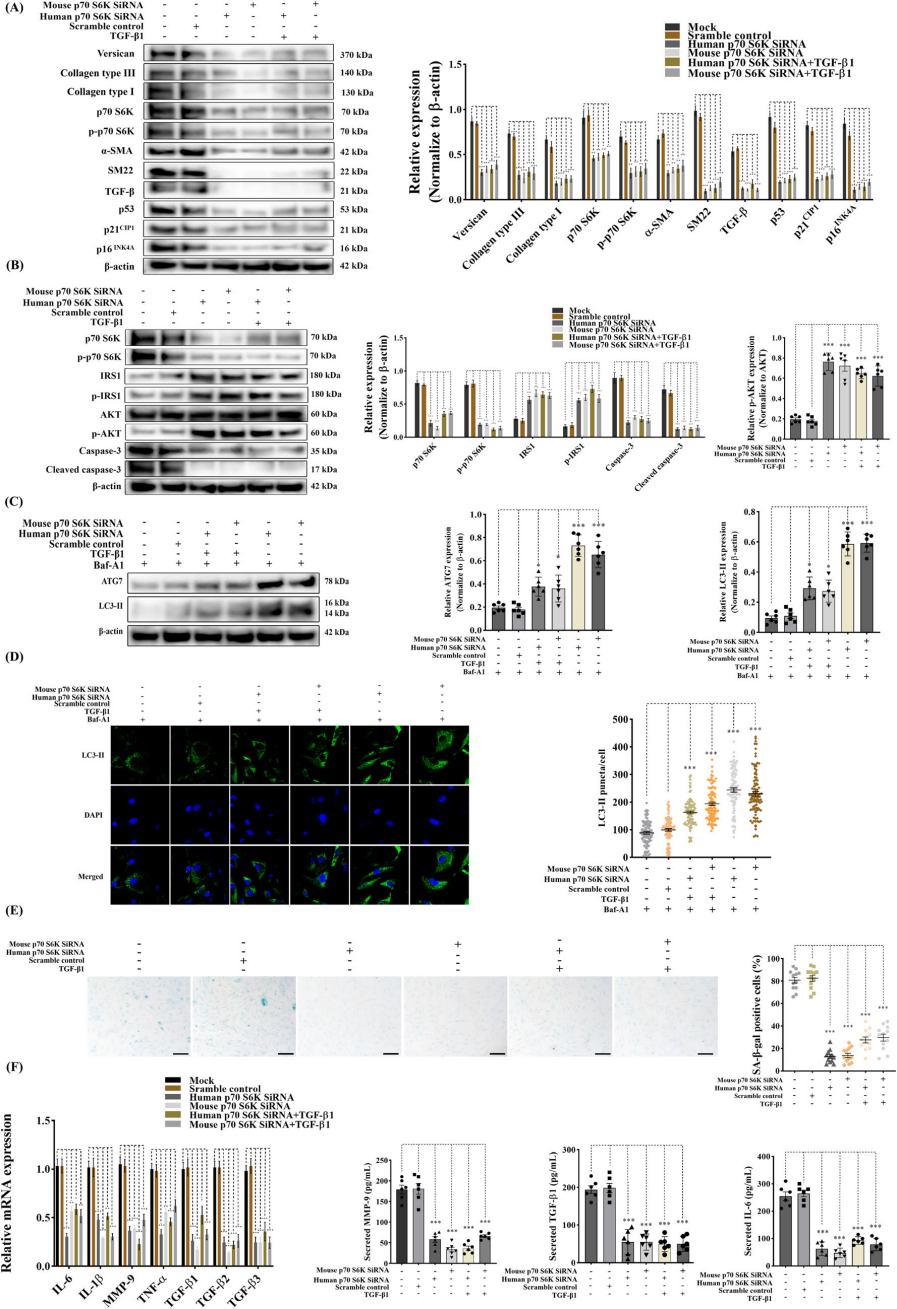
Knockdown of p70 S6K reverses VIC phenotype through upregulation of autophagy and inhibition of cellular senescence while protecting against apoptosis. Canine aVICs were transfected with Lipofectamine 3000 (Mock), scramble control, human p70 S6K siRNA, mouse p70 S6K siRNA with or without 10 ng/mL of TGF‐β1 or 5 μM of baflomycin‐A1 (Baf‐A1). (A) Representative western blot of p70 S6K, phospho‐p70 S6K (p‐p70 S6K), p16INK4A, p21CIP1 and p53, VIC phenotype and ECM protein expression and quantification of the relative protein expression (*n* = 6). (B) Representative western blot of p70 S6K, phospho‐p70 S6K (p‐p70 S6K), phospho‐IRS1 (p‐p70 IRS1), phospho‐AKT (p‐AKT), caspase‐3, cleaved caspase‐3 and quantification of the relative protein expression (*n* = 6) (C) Representative western blot of ATG7 and LC3‐II protein expression in qVICs treated with 5 μM baflomycin‐A1 with or without 10 ng/mL of TGF‐β1 and quantification of the relative protein expression (*n* = 6). (D) Representative confocal images of LC3‐II labelled autophagosomes (green) and quantitative analysis of the number of LC3‐II puncta, scale bar 20 μm (*n* = 98 cells/treatment). (E) Representative images of SA‐β‐gal (blue) staining and quantitative analysis of the percentage of SA‐β‐gal positive cells, scale bar 50 μm (*n* = 12 microscopic fields). (F) Quantitative RT‐PCR (left panel) for senescence‐associated secretory phenotype (SASP) cytokine expression in aVICs transfected with p70 S6K siRNA with or without TGF‐β induction (*n* = 6). Quantification of secreted TGF‐β1, IL‐6 and MMP‐9 (right panel) in collected supernatant from aVIC cultures (*n* = 6). Results are presented as mean ± SEM. ANOVA followed by Tukey's range test. *p < 0.05, **p < 0.01, ***p < 0.001 compared to control. ECM, extracellular matrix; VIC, valve interstitial cell.

To further examine the central regulatory effects of p70 S6K and its potential as a novel therapeutic target for MMVD, gene expression of p70 S6K was silenced using human and mouse siRNA in aVICs. The upregulation of α‐SMA and SM22‐α was dramatically attenuated by the downregulation of p70 S6K in aVICs in the presence or absence of TGF‐β1 treatment. As expected, the ECM protein synthesis (collagen type I, collagen type III, versican) and TGF‐β expression were significantly decreased with VIC phenotype recovery. In the presence of 10 ng/mL TGF‐β1, the knockdown of p70 S6K reduced the expression of the senescent transcription factors p16^INK4A^ and p53/p21^CIP1^ (Figure 10A), while there was a concurrent inactivation of caspase‐3 and cleaved caspase‐3 mediated apoptotic activities (Figure 10B), together with a significant increased capacity for BrdU incorporation (Figure S4B).

To further investigate the mechanism of unexpected decreased apoptosis, the phosphorylated forms of p70 S6K, IRS1 and AKT were examined considering their roles in the negative feedback loop from p70 S6K to IRS1 and the key regulatory functions of AKT in cell apoptosis though anti‐apoptotic proteins.^60^, ^61^ The decreased phosphorylated level of p70 S6K, caused by silencing p70 S6K, resulted in an increase in phosphorylated expression of IRS1 and the downstream effector AKT (Figure 10B). In the presence of 5 μM baflomycin‐A1, autophagy flux was promoted as shown by significantly increased expression of ATG7 and LC3‐II (Figure 10C). LC3‐II puncta were significantly increased by silencing p70 S6K (Figure 10D). p70 S6K downregulated aVICs showed less SA‐β‐gal staining (Figure 10E), more accumulation in S and G2/M phases (Figure S4A) and significant downregulation of SASP mRNA expression and the level of secreted IL‐6, MMP‐9, TGF‐β1 (Figure 10F). These data indicate that p70 S6K knockdown transitions cells to a more quiescent and normal phenotype, induces normal ECM homeostasis and abolishes cellular senescence and SASP, while inhibiting apoptosis and promoting a more autophagic state.

## 4 DISCUSSION

In this study, we have identified TGF‐β‐induced PI3K/AKT/mTOR/p70 S6K signalling controls mitral VIC differentiation, function and cellular activities in canine MMVD. A significantly increased expression of PI3K and TGF‐β was observed in α‐SMA positive aVICs in canine myxomatous mitral valves. Activation of PI3K/AKT/mTOR/p70 S6K signalling was shown to promote the transformation of activated myofibroblast. Pharmacological inhibition of PI3K signalling restored the normal quiescent VIC phenotype by suppressing senescence and SASP and promoting apoptosis and autophagy. Upregulation of mTOR/S6K induces transformation of senescent aVICs, with compromised apoptotic activity and impaired autophagy flux. Conversely, selective knockdown of p70 S6K reverses cell transition by attenuating cell senescence, inhibiting apoptosis and improving autophagy. These findings provide novel evidence that the PI3K signalling pathway antagonism is a promising target to inhibit the pathological processes of MMVD, by preventing myofibroblast transition, counteracting cellular senescence, SASP and restoring apoptosis and autophagy, with potential as a therapeutic target for MMVD in the dog, and by extension for the analogous human disease. Furthermore, the dysregulation of the PI3K/AKT/mTOR pathway in MMVD supports the concept that this degenerative disease is associated with tissue ageing.

Smad‐mediated canonical TGF‐β signalling has been shown to contribute to the pathogenesis of human MMVD, but to what extent this is driving fibrosis and/or myxomatous degeneration cannot be stated with certainty.^10^, ^11^, ^12^, ^16^ We have now shown that one of the non‐canonical pathways (PI3K) also likely contributes to MMVD pathogenesis. TGF‐βs are recognized as an important initiator of signalling pathways that contribute to the development of human and canine MMVD.^1^, ^62^ TGF‐β signalling‐dependent VIC phenotypic transformation and myxomatous degeneration are widely reported in both species.^10^, ^18^, ^63^ TGF‐β‐induced PI3K/AKT/mTOR activation has been widely reported in multiple degenerative disorders, including idiopathic pulmonary fibrosis (IPF), cardiac and renal fibrosis.^22^, ^64^, ^65^ The upregulation of PI3K/AKT/mTOR signalling in fibroblasts induces the aberrant transition of myofibroblastic phenotype and ECM remodelling, and therefore serves as a primary driver for the development and progression of these diseases. However, the role of these signalling pathways in both human and canine MMVD is not fully understood, in particular, the further downstream effects on transcription factors that control interstitial cell (VIC) phenotype, survival and ECM synthesis. To our knowledge, the current study is the first report showing that TGF‐β‐induced PI3K/Akt/mTOR signalling controls the phenotypical transitions of mitral VICs and their functional roles in ECM remodelling in MMVD. The PI3K/AKT/mTOR pathway is well characterized as an intracellular signalling pathway important in regulating cellular quiescence, differentiation and survival.^48^ We have shown that activation of PI3K/AKT/mTOR pathway promotes the transformation of mitral VICs into myofibroblasts and enhances the ECM protein synthesis. These data are consistent with observations in many other types of cells and diseases.^66^, ^67^, ^68^ The persistence of activated PI3K signalling depends on several regulatory mechanisms including AKT activation through a negative feedback loop from p70 S6K to PI3K and the upregulated phosphorylation of PRAS40.^43^, ^69^ In the present study, the high level of phosphorylated AKT, PRAS40, mTOR and p70 S6K observed in aVICs suggests AKT activation enhances PRAS40 phosphorylation and thereby reduces the inhibitory effects of PRAS40 on mTORC1, intensifying the mTOR/p70 S6K signalling pathway.^43^ Augmented mTOR/p70 S6K can inhibit the upstream IRS1 mediated signals initiating from other growth factor receptors rather than TGF‐β receptor I/II and thereby intensify the TGF‐β‐induced PI3K signalling.^69^ This could explain the persistence and survival of aVICs resulting from aberrantly activated PI3K/Akt/mTOR/p70 S6K signalling in the development of MMVD. However, other growth factors, such as insulin, fibroblast growth factor (FGF), insulin‐like growth factor (IGF) and epidermal growth factor (EGF), have been shown to also trigger PI3K/AKT/mTOR signalling.^70^, ^71^ Furthermore, the antagonism of canonical Smad2/3‐mediated TGF‐β signalling has similar effects on mitral VIC transformation and ECM remodelling.^10^ To what extent one of these might be a dominant pathway for the disease, and the interplay between these signalling pathways, are still unknown and requires further study.

As a direct downstream target of PI3K, AKT is at the molecular junction controlling cell death and survival. AKT promotes cell survival by blocking apoptosis through the inactivation of pro‐apoptotic proteins such as Bcl‐2.^60^ In our study, AKT inactivation by pharmacological inhibition of PI3K signalling induced cell apoptosis in aVICs. This may in part explain aVIC persistence in MMVD.^72^ As a form of programmed cell death, apoptosis is a key factor causing target cells to be cleared from tissues. However, VICs play a crucial role in the maintenance of a balanced ECM in mitral valves and directly removing them may also result in further damage to the ECM.^73^, ^74^ In the current study inhibiting the downstream AKT effectors mTOR/S6K by selective silencing p70 S6K improved autophagy flux and attenuated cell senescence and SASP, while inhibiting apoptosis. Considering the apoptosis inhibition induced by AKT activation through the negative feedback loop between mTOR/p70 S6K and IRS1/AKT,^60^, ^61^ selectively targeting the downstream effector mTOR may be a better way to restore cell transitions and functions, minmising undesirable effects.

Recently mTOR/p70 S6K signalling has received wide attention due to its key roles in controlling cell transition, apoptosis, autophagy and senescence. Small‐molecule modulators based on the manipulation of mTOR have been investigated in a variety of cardiovascular diseases.^75^ Activation of mTOR is known to result in myofibroblast differentiation, inhibition of apoptosis and autophagy while enhancing cellular senescence. This is consistent with our observations where we have induced p70 S6K overexpression qVICs. Conversely, inhibition of mTOR signalling in aVICs by global pharmacological manipulation of PI3K signalling or selective knockdown of p70 S6K reversed cell phenotype, revived ECM protein synthesis and promoted apoptosis and autophagy, while reversing cell senescence. This suggests a pivotal role for mTOR/p70 S6K signalling in the regulation of important VIC activities in MMVD. p70 S6K has been shown to regulate mRNA translation initiation and thereby protein synthesis.^75^ Increased α‐SMA expression induced by activation of mTOR/p70 S6K signalling has been observed in pulmonary artery smooth muscle cells (SMCs), while the mTOR inhibitor rapamycin suppressed the proliferation of α‐SMA positive SMCs.^76^ In addition, the accumulation of SM22 in myocytes with a contractile phenotype can be reduced by PI3K/mTOR/p70 S6K inhibition by LY294002 and rapamycin treatment.^77^ This again is consistent with our observations in VIC transition being regulated by PI3K signalling in MMVD. On that basis, it would be reasonable to presume that p70 S6K regulates the function and differentiation of VICs through the phosphorylation of its substrate S6 ribosomal protein, and this might be considered as a potential therapeutic target. Pharmacological inhibition or knockdown of mTOR/p70 S6K has been shown to protect against human intervertebral disc apoptosis, cellular senescence and ECM catabolism, through autophagy induction.^24^, ^25^ Mice with hypomorphic mTOR have increased lifespan and reduced senescent marker p16^INK4A^ expression, demonstrating the link between tissue degeneration and ageing, while mice with articular cartilage‐specific mTOR deletion are protected against osteoarthritis through enhancement of autophagy.^49^ The complex interplay between these cellular and molecular responses needs to be further investigated.

In the present study, the typical characteristics of cellular senescence were noticeable in aVICs as shown by decreased apoptotic activities, nuclear γ‐H2AX foci formation, cytoplasmic positive SA‐β‐gal staining, activation of p53/p21^CIP1^ and p16^INK4A^ pathways, and increased intracellular and extracellular SASP expression. Senescence is now considered to be a highly dynamic process associated with multiple cellular, molecular changes and distinct phenotypic alterations. Senescent cells resist elimination from tissues by apoptosis through the upregulation of anti‐apoptotic pathways.^52^ Considering the activation of PI3K signalling in aVICs and its effects on promoting cell proliferation and transformation, we can reasonably speculate that due to PI3K activation some transformed aVICs can undergo senescent changes while others retain the proliferative ability, and so contribute to more senescent aVICs as MMVD develops and progresses. Since senescent cells remain metabolically active, despite being in a growth‐arrested state, they will affect the events inside cells, the behaviour of neighbouring non‐senescent cells and the remodelling of the surrounding microenvironment by secreting a complex mixture of secreted factors (SASP). This special secretome includes inflammatory, pro‐apoptotic, insulin resistance‐inducing cytokines, such as IL‐6, IL‐1β and TNF‐α, MMPs and relevant regulators such as MMP‐9 and TIMP1 that cause ECM remodelling, and finally TGF‐β family members that contribute to fibrosis, myxomatous degeneration and dysregulated trans‐differentiation of VICs.^52^ In the current study, the accumulation of senescent cells and expression of SASP suggests that as the disease develops TGF‐β‐induced activation of PI3K/AKT/mTOR/p70 S6K signalling triggers the proliferation and abnormal transition of qVICs to senescent aVICs with reduced ability of apoptosis and autophagy. Subsequent induction of SASP formation intensifies TGF‐β signalling, causing abnormal ECM remodelling and continual valve tissue damage. SASP itself may be caused, in part, by senescence‐associated mitochondrial dysfunction (SAMD), closely associated with the dysregulation of mTOR/p70 S6K signalling. Increased pro‐inflammatory cytokines (IL‐6, IL‐1β and TNF‐α) can be induced by activated danger‐associated molecular patterns, such as reactive oxygen species and mtDNA fragments, released by dysfunctional mitochondria, which may then contribute to ECM damage.^78^ Further studies are required to examine the possible contribution of mitochondrial dysfunction to MMVD pathogenesis.

In targeting the highly expressed Senescent Cell Anti‐Apoptotic Pathway networks, senolytic compounds have been shown to clear senescent cells by promoting apoptosis through the upregulation of multiple pro‐apoptotic pathways.^79^ The naturally occurring flavonoid quercetin has been reported to remove senescent cells through apoptosis by inhibiting PI3K/AKT/mTOR signalling, in a similar fashion to LY294002, copanlisib and alpelisib used in this study.^80^ Flavonoids might serve as better potential therapeutic candidates to treat MMVD due to their wide availability and moderate toxicity. However, clearance of functional VICs in myxomatous valves may cause unexpected valvular outcomes and it is likely reversing aVICs to a non‐senescent qVICs using autophagy activators inhibiting mTOR/p70 S6K signalling might be a more promising therapeutic approach to control MMVD in both the human and the dog.^75^

The findings of this study will require in vivo validation. Although in vitro primary cells isolated from clinical samples are largely able to reflect the disease parthenogenesis, in vivo validations are needed considering the drug metabolism, individual variance and clinical transitional research. However, there are a limited numbers of animal models available to study the pathogenesis of MMVD. A recently developed spontaneously occurring FVB/NJ mouse model of MMVD rapidly exhibits disease progression and pathology and would appear to be a good candidate.^81^ Specifically, to further validate whether PI3K/AKT/mTOR signalling controls the MMVD progression in vivo, a novel genetically modified VIC‐specific p70 S6K knockout or overexpression mouse model is required.

**FIGURE 11:**
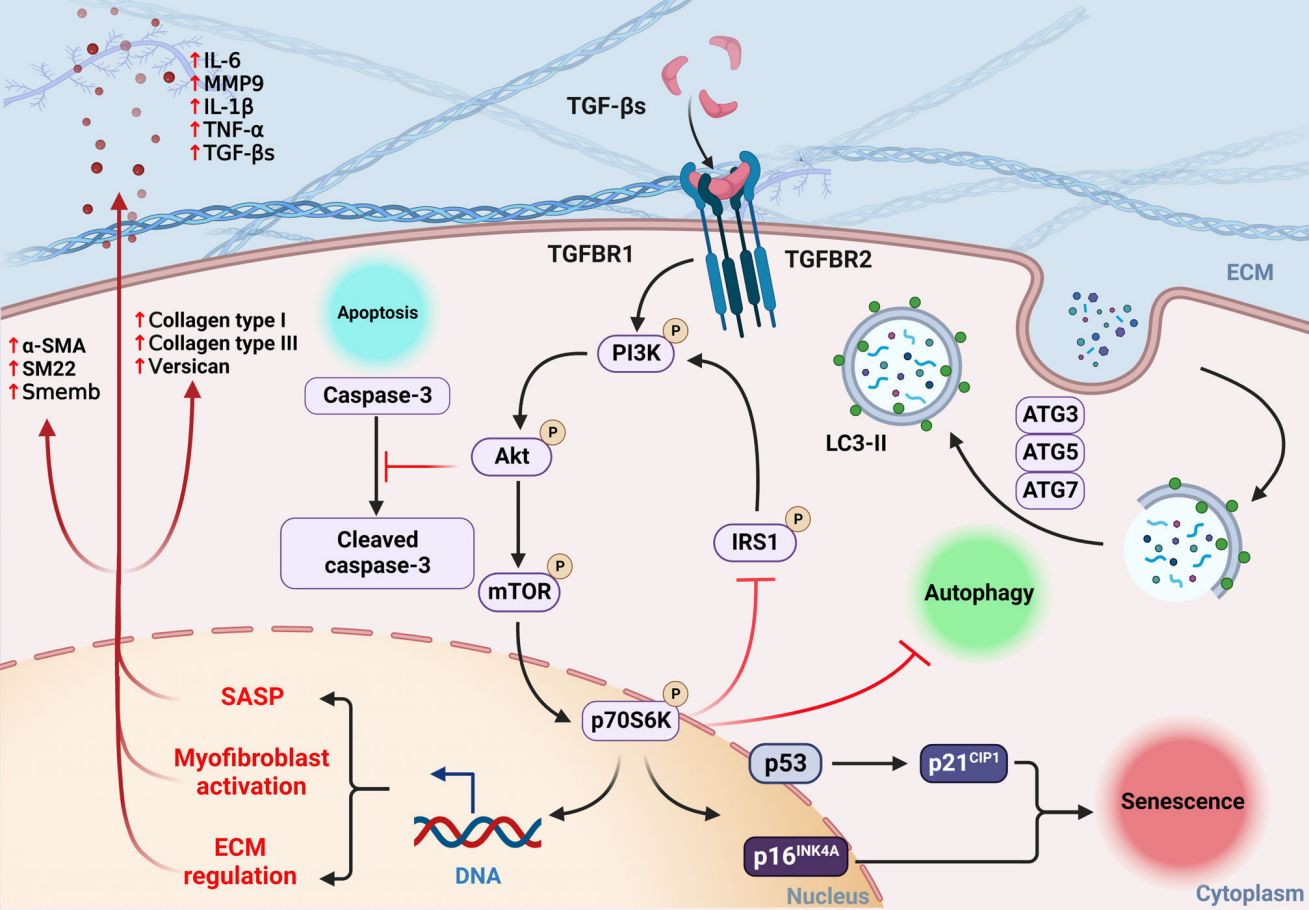
Schematic illustration of TGF‐β‐induced PI3K/AKT/mTOR/p70 S6K pathway in the regulation of cell phenotype, ECM synthesis, apoptosis, autophagy and senescence in aVICs in canine MMVD. aVIC, activated myofibroblast phenotype; ECM, extracellular matrix; MMVD, myxomatous mitral valve disease; TGF‐β, transforming growth factor β.

In conclusion, TGF‐β‐induced PI3K/AKT/mTOR/p70 S6K signalling controls the phenotypic transformation and functions of VICs in canine MMVD (Figure 11). Pharmacological inhibition of PI3K signalling reverses diseased senescent VICs, with improved capacity for apoptosis and autophagy. Furthermore, downstream mTOR/p70 S6K signalling plays an important role in the regulation of VIC transformation, ECM protein synthesis, apoptosis, autophagy and senescence in MMVD. This work informs the naturally occurring disease in dogs as a novel large animal model to investigate human early stage MMVD and warrants further investigations of senolytic compounds or autophagy activators as a potential novel therapeutic strategy for the treatment of MMVD and other age‐related degenerative disorders.

## AUTHOR CONTRIBUTIONS

Qiyu Tang, Kanchan Phadwal, Vicky E. MacRae and Brendan M. Corcoran conceptualized the study and and experiments. Qiyu Tang, Greg R. Markby, Andrew J. MacNair, Keyi Tang and Michal Tkacz performed the experiments. Qiyu Tang and Kanchan Phadwal accomplished data analysis and prepared the manuscript. Maciej Parys, Kanchan Phadwal, Vicky E. MacRae and Brendan M. Corcoran evaluated the manuscript and data reliability. All authors have read and agreed to the published version of the manuscript.

## CONFLICT OF INTEREST STATEMENT

The authors declare no conflict of interest.

## Supporting information


**DATA S1.** Supporting Information

## Data Availability

The present research makes no reference to publicly accessible or shareable data. All original data that support the conclusions of this work are available from the corresponding author upon reasonable request.
