# Supplementary material for: TGF‐β‐induced PI3K/AKT/mTOR pathway controls myofibroblast differentiation and secretory phenotype of valvular interstitial cells through the modulation of cellular senescence in a naturally occurring in vitro canine model of myxomatous mitral valve disease
Source: Cell Prolif. 2023 Mar 4;56(6):e13435. doi: 10.1111/cpr.13435 (PMC10280140; doi:10.1111/cpr.13435)
Supplement: Supplementary file 1 — DATA S1. Supporting Information [file CPR-56-e13435-s001.docx]

**Supplementary Table 1. siRNA sequences for gene silencing.**

| siRNA duplex | Sense sequence (5ʹ-3ʹ) | Antisense sequence (5ʹ-3ʹ) |
| --- | --- | --- |
| Human p70 S6K siRNA 1 | CAAGGUCAUGUGAAACUAAtt | UUAGUUUCACAUGACCUUGtt |
| Human p70 S6K siRNA 2 | GAGAGUCAAUGUCAUUACAtt | UGUAAUGACAUUGACUCUCtt |
| Mouse p70 S6K siRNA 1 | CAUGGAACAUUGUGAGAAAtt | UUUCUCACAAUGUUCCAUGtt |
| Mouse p70 S6K siRNA 2 | GGAAGAUAUUUGCCAUGAAtt | UUCAUGGCAAAUAUCUUCCtt |
| Mouse p70 S6K siRNA 3 | CGUGAACAAUUCUGUAGUAtt | UACUACAGAAUUGUUCACGtt |

**Supplementary Table 2. cDNA ORF sequences for gene overexpression.**

| **Human p70 S6K cDNA ORF clone** |
| --- |
| ATGAGGCGAC GAAGGAGGCG GGACGGCTTT TACCCAGCCC CGGACTTCCG AGACAGGGAA GCTGAGGACA TGGCAGGAGT GTTTGACATA GACCTGGACC AGCCAGAGGA CGCGGGCTCT GAGGATGAGC TGGAGGAGGG GGGTCAGTTA AATGAAAGCA TGGACCATGG GGGAGTTGGA CCATATGAAC TTGGCATGGA ACATTGTGAG AAATTTGAAA TCTCAGAAAC TAGTGTGAAC AGAGGGCCAG AAAAAATCAG ACCAGAATGT TTTGAGCTAC TTCGGGTACT TGGTAAAGGG GGCTATGGAA AGGTTTTTCA AGTACGAAAA GTAACAGGAG CAAATACTGG GAAAATATTT GCCATGAAGG TGCTTAAAAA GGCAATGATA GTAAGAAATG CTAAAGATAC AGCTCATACA AAAGCAGAAC GGAATATTCT GGAGGAAGTA AAGCATCCCT TCATCGTGGA TTTAATTTAT GCCTTTCAGA CTGGTGGAAA ACTCTACCTC ATCCTTGAGT ATCTCAGTGG AGGAGAACTA TTTATGCAGT TAGAAAGAGA GGGAATATTT ATGGAAGACA CTGCCTGCTT TTACTTGGCA GAAATCTCCA TGGCTTTGGG GCATTTACAT CAAAAGGGGA TCATCTACAG AGACCTGAAG CCGGAGAATA TCATGCTTAA TCACCAAGGT CATGTGAAAC TAACAGACTT TGGACTATGC AAAGAATCTA TTCATGATGG AACAGTCACA CACACATTTT GTGGAACAAT AGAATACATG GCCCCTGAAA TCTTGATGAG AAGTGGCCAC AATCGTGCTG TGGATTGGTG GAGTTTGGGA GCATTAATGT ATGACATGCT GACTGGAGCA CCCCCATTCA CTGGGGAGAA TAGAAAGAAA ACAATTGACA AAATCCTCAA ATGTAAACTC AATTTGCCTC CCTACCTCAC ACAAGAAGCC  AGAGATCTGC TTAAAAAGCT GCTGAAAAGA AATGCTGCTT CTCGTCTGGG AGCTGGTCCT GGGGACGCTG GAGAAGTTCA AGCTCATCCA TTCTTTAGAC ACATTAACTG GGAAGAACTT CTGGCTCGAA AGGTGGAGCC CCCCTTTAAA CCTCTGTTGC AATCTGAAGA GGATGTAAGT CAGTTTGATT CCAAGTTTAC ACGTCAGACA CCTGTCGACA GCCCAGATGA CTCAACTCTC AGTGAAAGTG CCAATCAGGT CTTTCTGGGT TTTACATATG TGGCTCCATC TGTACTTGAA AGTGTGAAAG AAAAGTTTTC CTTTGAACCA AAAATCCGAT CACCTCGAAG ATTTATTGGC AGCCCACGAA CACCTGTCAG CCCAGTCAAA TTTTCTCCTG GGGATTTCTG GGGAAGAGGT GCTTCGGCCA GCACAGCAAA TCCTCAGACA CCTGTGGAAT ACCCAATGGA AACAAGTGGC  ATAGAGCAGA TGGATGTGAC AATGAGTGGG GAAGCATCGG CACCACTTCC AATACGACAG CCGAACTCTG GGCCATACAA AAAACAAGCT TTTCCCATGA TCTCCAAACG GCCAGAGCAC CTGCGTATGA ATCTA |
| **Mouse p70 S6K cDNA ORF clone** |
| ATGAGGCGAC GACGGAGGCG GGACGGCTTT TACCTAGCGC CTGACTTCCG ACACAGGGAA GCTGAGGACA TGGCAGGAGT GTTTGACATA GACCTGGACC AGCCAGAAGA TGCAGGCTCT GAGGATGAGC TGGAGGAGGG GGGTCAGTTA AATGAAAGCA TGGACCATGG GGGAGTTGGA CCATATGAAC TTGGCATGGA ACATTGTGAG AAATTTGAAA TCTCAGAAAC TAGTGTGAAC AGAGGGCCAG AAAAAATCAG ACCAGAATGT TTTGAGCTAC TTCGGGTACT TGGTAAAGGG GGCTATGGAA AGGTTTTTCA AGTACGAAAA GTAACAGGAG CAAATACTGG GAAGATATTT GCCATGAAGG TGCTTAAAAA GGCAATGATA GTGAGGAATG CTAAGGACAC GGCCCACACG AAAGCAGAGC GGAACATTCT GGAGGAAGTG AAACACCCTT TCATTGTGGA CCTGATTTAT GCCTTTCAGA CCGGAGGAAA GCTCTACCTC ATCCTCGAGT ATCTCAGTGG AGGAGAACTA TTTATGCAGT TAGAAAGAGA GGGAATATTC ATGGAAGACA CAGCGTGCTT TTACTTGGCT GAAATCTCCA TGGCTTTGGG GCATTTACAT CAAAAAGGGA TCATCTACAG AGACCTGAAG CCGGAGAACA TCATGCTTAA TCACCAAGGT CACGTGAAAC TAACAGACTT TGGACTATGC AAAGAATCTA TTCATGATGG AACAGTCACG CACACATTTT GTGGAACAAT AGAATACATG GCCCCTGAAA TCTTAATGAG AAGCGGCCAC AACCGTGCTG TGGATTGGTG GAGTTTGGGA GCATTAATGT ATGACATGCT GACTGGAGCA CCTCCATTCA CTGGGGAGAA TAGAAAGAAA ACAATTGACA AAATCCTCAA ATGTAAACTT AATTTGCCTC CCTACCTCAC ACAAGAAGCT CGAGATCTGC TTAAAAAGCT GCTGAAAAGA AATGCTGCTT CTCGTCTTGG AGCTGGCCCT GGGGATGCTG GAGAAGTCCA AGCTCATCCT TTTTTCAGAC ACATTAACTG GGAAGAACTT CTGGCTCGGA AGGTGGAACC TCCCTTTAAG CCTCTGTTGC AATCTGAAGA GGATGTGAGT CAGTTTGATT CAAAGTTTAC ACGTCAGACA CCTGTTGACA GCCCTGATGA CTCCACTCTC AGTGAAAGTG CCAACCAGGT CTTTCTGGGT TTTACATATG TGGCTCCATC TGTACTTGAA AGTGTGAAAG AAAAGTTTTC ATTTGAACCA AAAATCCGAT CTCCTAGAAG ATTTATTGGT AGTCCACGAA CACCTGTCAG CCCAGTCAAA TTCTCTCCTG GGGATTTCTG GGGACGAGGT GCTTCAGCCA GCACGGCAAA TCCTCAGACC CCTGTGGAAT ACCCAATGGA AACAAGTGGG ATAGAGCAGA TGGATGTGAC AGTGAGCGGG GAAGCATCAG CGCCACTTCC AATCCGACAA CCCAACTCCG GGCCGTACAA AAAACAAGCT TTTCCTATGA TCTCCAAACG GCCAGAGCAC CTGCGGATGA ATCTA |

**Supplementary Table 3. Antibodies and reagents used in this study.**

| **Host** | **Company** | **Antigen** | **Catalog number** |
| --- | --- | --- | --- |
| Rabbit | Cell Signaling Technology | PI3K p110α | #4249 |
| Rabbit | Antibodies | PI3K p110α | A94027 |
| Rabbit | Cell Signaling Technology | Akt | #4685 |
| Rabbit | Cell Signaling Technology | phosphorylated Akt (Ser473) | #4060 |
| Rabbit | Cell Signaling Technology | mTOR | #2983 |
| Rabbit | Cell Signaling Technology | phosphorylated mTOR (Ser2448) | #5536 |
| Rabbit | Cell Signaling Technology | p70 S6K | #9202 |
| Rabbit | Cell Signaling Technology | phosphorylated p70 S6K (Thr389) | #9234 |
| Rabbit | Cell Signaling Technology | PRAS40 | #2691 |
| Rabbit | Cell Signaling Technology | phosphorylated PRAS40 (Thr246) | #2997 |
| Rabbit | Cell Signaling Technology | IRS1 | #2382 |
| Rabbit | Cell Signaling Technology | phosphorylated IRS1 (Ser636/639) | #2388 |
| Rabbit | Cell Signaling Technology | β-actin | #8457 |
| Rabbit | Proteintech | p16^INK4A^ | 10883-1-AP |
| Rabbit | Proteintech | p21^CIP1^ | 10355-1-AP |
| Rabbit | Proteintech | p53 | 60283-2-Ig |
| Rabbit | Cell Signaling Technology | caspase-3 | #9662 |
| Rabbit | Cell Signaling Technology | cleaved caspase-3 | #9661 |
| Rabbit | Abcam | ATG3 | ab108251 |
| Rabbit | Cell Signaling Technology | ATG5 | #12994 |
| Rabbit | Abcam | ATG7 | ab52472 |
| Rabbit | Cell Signaling Technology | α-SMA | #19245 |
| Rabbit | MBL | LC3 | PM036 |
| Rabbit | Proteintech | SM22 | 10493-1-AP |
| Rabbit | Abcam | versican | ab177480 |
| Rabbit | Proteintech | collagen type I | 14695-1-AP |
| Rabbit | Proteintech | collagen type III | 22734-1-AP |
| Rabbit | Proteintech | TGF-β 1 | 21898-1-AP |
| Mouse | R&D system | TGF-β 1, 2, 3 | MAB1835 |
| Mouse | Cell Signaling Technology | α-SMA | #48938 |
| Mouse | Abcam | MMP-9 | ab58803 |
| Mouse | Proteintech | p21^CIP1^ | 67362-1-Ig |
| Mouse | Proteintech | ATG7 | 67341-1-Ig |
| Goat | Dako | Anti-mouse secondary antibody | P0047 |
| Goat | Dako | anti-rabbit secondary antibody | P0048 |
|  | Cayman Chemical | LY294002 | 70920 |
|  | Cayman Chemical | Copanlisib | 20354 |
|  | Cayman Chemical | Alpelisib | 16986 |
|  | Cayman Chemical | Bafilomycin A1 | 11038 |
|  | R&D system | SC-79 | 4635 |
|  | Sino Biological | TGF-β1 | 10804-HNAC |
|  | Vector Labs | Hematoxylin | H-3401-500 |
|  | Vector Labs | 3,3’-diaminobenzidine (DAB) | SK-4100 |

**Supplementary Table 4. The qPCR primer sequences for target genes.**

| **Gene** | **Forward primer (5ʹ-3ʹ)** | **Reverse primer (5ʹ-3ʹ)** |
| --- | --- | --- |
| *ACTA2*  (α-SMA) | GGGGATGGGACAAAAGGACA | GCCACGTAGCAGAGCTTCTCCTTGA |
| TAGLN (SM22) | AAGAACGGCGTGATTCTGAG | CGGTAGTGCCCATCATTCTT |
| MYH10 (Smemb) | AGAAGCGAGCTGGAAAACTG | TCTTGCTCTGTCCGATTCTG |
| CASP3  (Caspase-3) | GCGGAAACCCACGGGGTTCG | CGGATGCGAGCCCGGGAAAG |
| GAPDH | ATCACTGCCACCCAGAAGAC | TCAGCTCAGGGATGACCTTG |
| IL-6 | TTAAGTACATCCTCGGCAAAATCT | CAGTGCCTCTTTGCTGTCTTCA |
| IL-1β | CAAGTCTCCCACCAGCTCTGTA | GGGCTTCTTCAGCTTCTCCAA |
| TNF-α | TCTCGAACCCCAAGTGACAAG | CAACCCATCTGACGGCACTA |
| MMP-9 | TGAGAACTAATCTCACTGACAAGCA | GCTCGGCCACTTGAGTGTA |
| TGF-β1 | CAAGGATCTGGGCTGGAAGTGGA | CCAGGACCTTGCTGTACTGCGTGT |
| TGF-β2 | GCAGCAAGACGATAATCACG | TCTTGTCGCTGTCGTCCTC |
| TGF-β3 | CTGGCCCTTTACAACAGCAC | CGACTCGGTGTTTTCCTGAG |

**
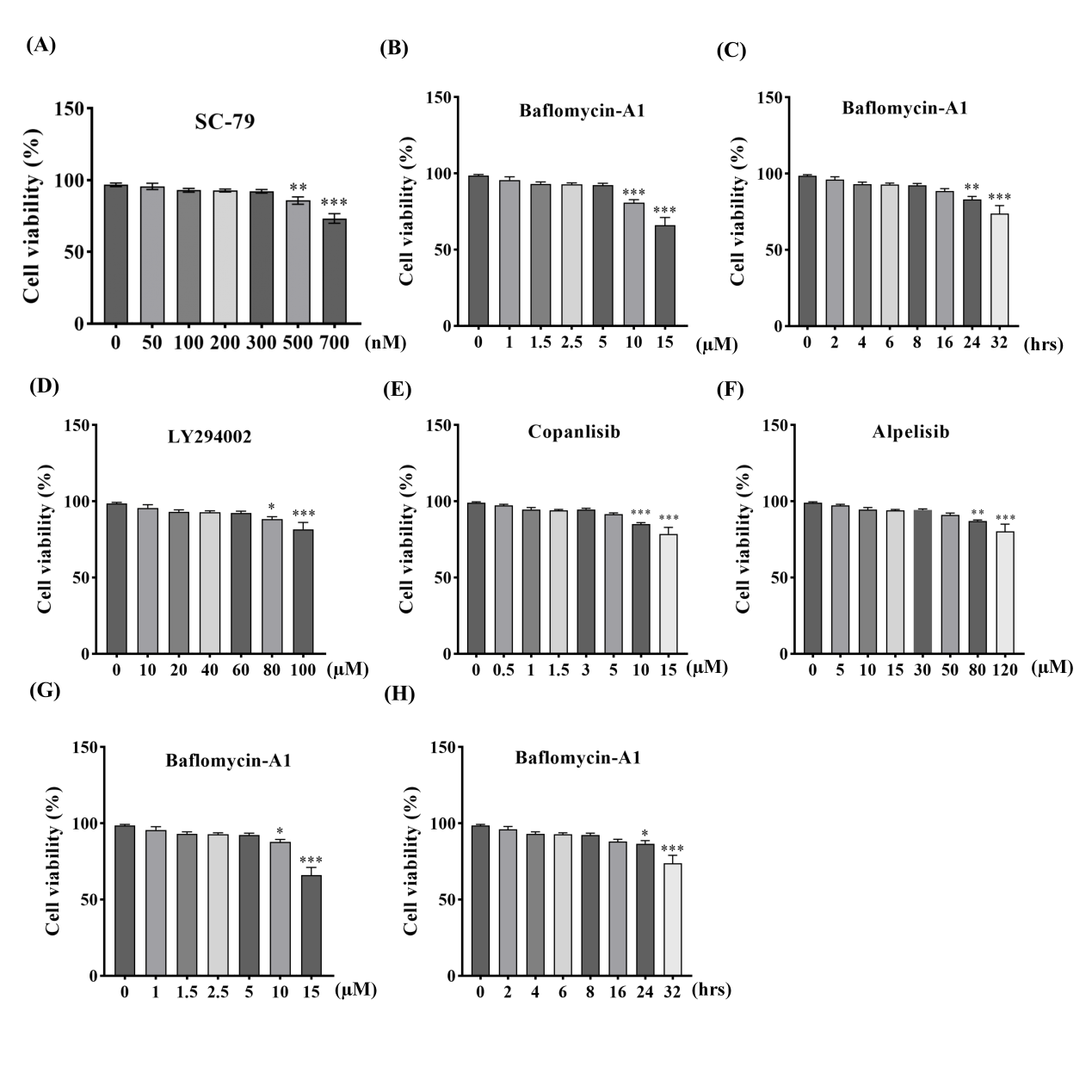
**

**Supplementary Figure 1. Cell viability assays for LY294002, copanlisib, alpelisib, SC-79 and baflomycin-A1.** Cell viability evaluation was examined by alamarBlue analysis. (A, B and C) Quantitative analysis for healthy qVIC viability. (D, E, F, G and H) Quantitative analysis for diseased aVIC viability. Results are presented as mean ± SEM. ANOVA followed by Tukey's range test. **p*< 0.05, ***p*< 0.01, ****p*< 0.001 compared to control.


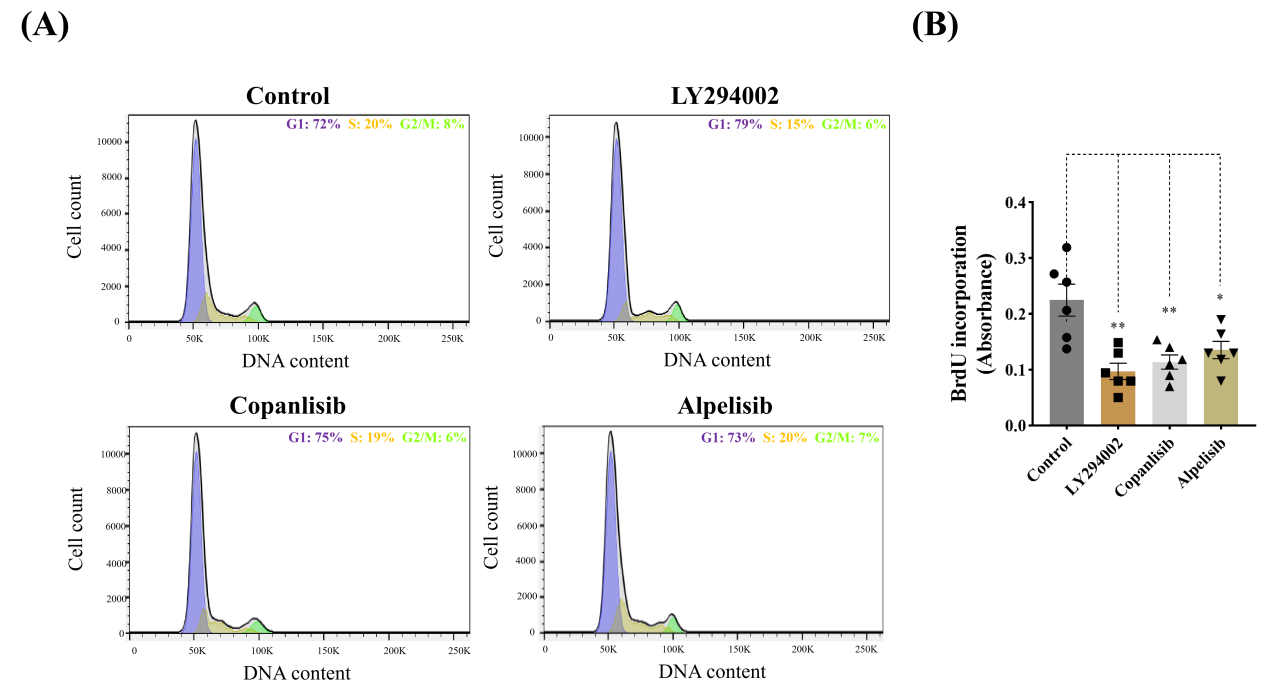


**Supplementary Figure 2. Pharmacological inhibition of PI3K signaling inhibits aVIC proliferative potential.** aVICs were exposed to DMSO (Control), LY294002 (60 µM), copanlisib (5 µM) and alpelisib (50 µM) treatment for 24 h. (A) Cell cycle analysis of canine aVICs treated with DMSO, LY294002 (60 µM), copanlisib (5 µM) and alpelisib (50 µM), followed by DNA staining by Hoechst 33342 (n=3). (B) BrdU incorporation assay of canine aVICs treated with DMSO, LY294002 (60 µM), copanlisib (5 µM) and alpelisib (50 µM) (n=6). Results are presented as mean ± SEM. ANOVA followed by Tukey's range test. **p*<0.05, ***p*<0.01, ****p*<0.001 compared to control.

**
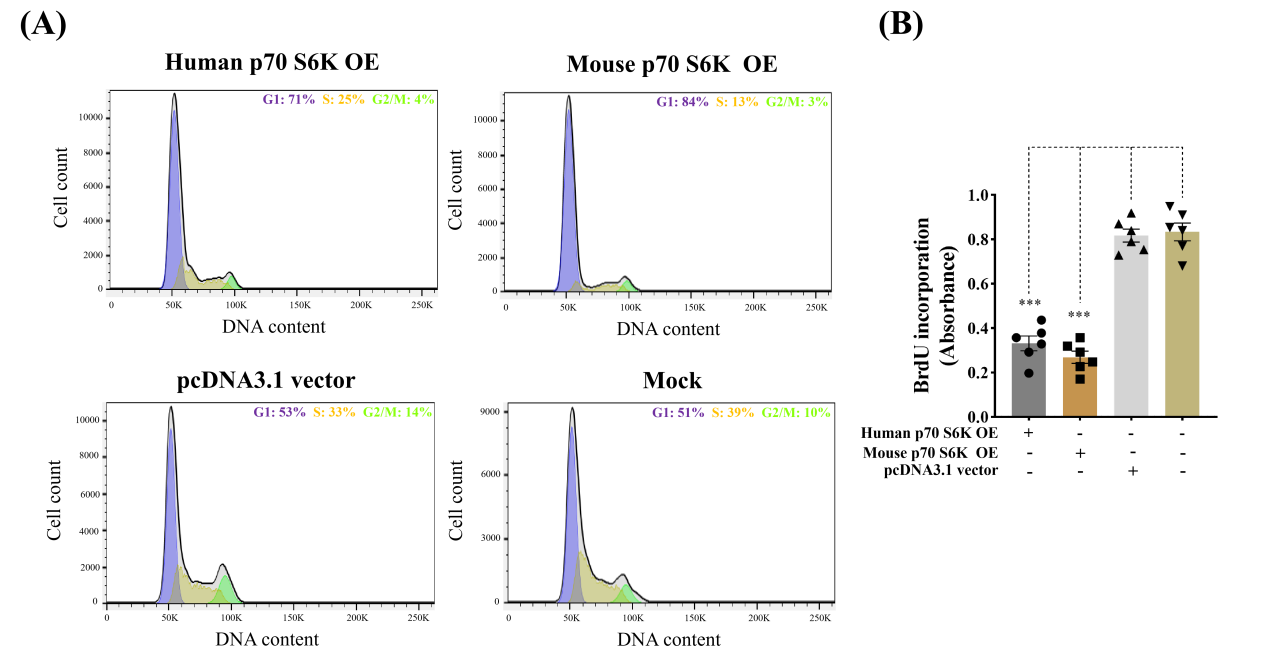
**

**Supplementary Figure 3. Overexpression of p70 S6K in qVICs induces G1 arrest and reduces cell proliferation.** Canine qVICs were transfected with human p70 S6K cDNA ORF plasmids, mouse p70 S6K cDNA ORF plasmids, pcDNA3.1 plasmids (vectors) and Lipofectamine 3000 (Mock). (A) Cell cycle analysis of canine qVICs transfected with p70 S6K cDNA ORF plasmids, vectors and Lipofectamine 3000 (n=3). (B) BrdU incorporation assay of canine qVICs transfected with p70 S6K cDNA ORF plasmids, vectors and Lipofectamine 3000 (n=6). Results are presented as mean ± SEM. ANOVA followed by Tukey's range test. **p*<0.05, ***p*<0.01, ****p*<0.001 compared to control.

**
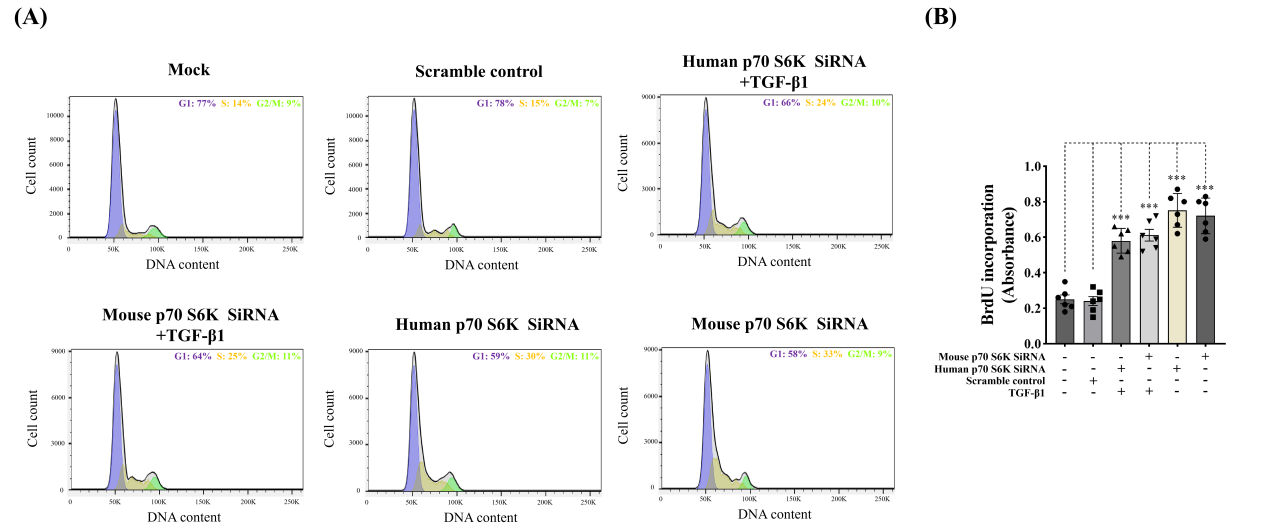
**

**Supplementary Figure 4. p70 S6K knockdown alleviates G1 arrest and increases cell proliferation in aVICs.** Canine aVICs were transfected with Lipofectamine 3000 (Mock), scramble control, human p70 S6K siRNA, mouse p70 S6K siRNA with or without 10 ng/mL of TGF-β1. (A) Cell cycle analysis of canine aVICs transfected with Lipofectamine 3000 (Mock), scramble control, human p70 S6K siRNA, mouse p70 S6K siRNA with or without 10 ng/mL of TGF-β1 (n=3). (B) BrdU incorporation assay of canine aVICs transfected with Lipofectamine 3000 (Mock), scramble control, human p70 S6K siRNA, mouse p70 S6K siRNA with or without 10 ng/mL of TGF-β1 (n=6). Results are presented as mean ± SEM. ANOVA followed by Tukey's range test. **p*<0.05, ***p*<0.01, ****p*<0.001 compared to control.
